# Fuzzy interactions between the auto-phosphorylated C-terminus and the kinase domain of CK1δ inhibits activation of TAp63α

**DOI:** 10.1038/s41598-023-43515-x

**Published:** 2023-09-30

**Authors:** Mahil Lambert, Jakob Gebel, Charlotte Trejtnar, Nicole Wesch, Süleyman Bozkurt, Martin Adrian-Allgood, Frank Löhr, Christian Münch, Volker Dötsch

**Affiliations:** 1 Institute of Biophysical Chemistry and Center for Biomolecular Magnetic Resonance, Goethe University, Frankfurt/Main, Germany; 2 Institute of Biochemistry II, Faculty of Medicine, Goethe University, Frankfurt/Main, Germany; 3 Frankfurt Cancer Institute, Frankfurt/Main, Germany; 4 Cardio-Pulmonary Institute, Frankfurt/Main, Germany

**Keywords:** Biochemistry, Biophysical chemistry

## Abstract

The p53 family member TAp63α plays an important role in maintaining the genetic integrity in oocytes. DNA damage, in particular DNA double strand breaks, lead to the transformation of the inhibited, only dimeric conformation into the active tetrameric one that results in the initiation of an apoptotic program. Activation requires phosphorylation by the kinase CK1 which phosphorylates TAp63α at four positions. The third phosphorylation event is the decisive step that transforms TAp63α into the active state. This third phosphorylation, however, is ~ 20 times slower than the first two phosphorylation events. This difference in the phosphorylation kinetics constitutes a safety mechanism that allows oocytes with a low degree of DNA damage to survive. So far these kinetic investigations of the phosphorylation steps have been performed with the isolated CK1 kinase domain. However, all CK1 enzymes contain C-terminal extensions that become auto-phosphorylated and inhibit the activity of the kinase. Here we have investigated the effect of auto-phosphorylation of the C-terminus in the kinase CK1δ and show that it slows down phosphorylation of the first two sites in TAp63α but basically inhibits the phosphorylation of the third site. We have identified up to ten auto-phosphorylation sites in the CK1δ C-terminal domain and show that all of them interact with the kinase domain in a “fuzzy” way in which not a single site is particularly important. Through mutation analysis we further show that hydrophobic amino acids following the phosphorylation site are important for a substrate to be able to successfully compete with the auto-inhibitory effect of the C-terminal domain. This auto-phosphorylation adds a new layer to the regulation of apoptosis in oocytes.

## Introduction

The genetic integrity of germ cells is of paramount importance for the survival of species and consequently, quality surveillance systems have evolved already early during evolution^1–3^. One of the proteins that is involved in eliminating oocytes with DNA double strand breaks is p63^4,5^, a member of the p53 family of transcription factors^6^. In mammals, oocytes spend most of their time in a quiescent state before meiosis I is completed and therefore contain a fourfold set of the genetic information. While this situation allows for efficient DNA repair via the process of homologous recombination^7,8^ the high DNA content makes oocytes also vulnerable to DNA damage. The challenge during evolution was to develop a surveillance system that effectively detects and eliminates oocytes with severe DNA damage to protect the genetic integrity but that is triggered only above a certain threshold level of damage. While many processes in oocytes are downregulated, DNA can get damaged, for example, by the remaining metabolism creating reactive molecules or by background ionizing radiation. A too sensitive trigger level for oocyte cell death would therefore endanger the survival of the species by eliminating oocytes before reproduction occurs. Several components contribute to the tight regulation of the apoptotic program in oocytes. First, TAp63α, the central transcription factor that upregulates the apoptotic program in oocytes^4^, adopts in resting primary oocytes an inactive state by forming a closed, only dimeric conformation^9^. This conformation is stabilized by interaction between the C-terminal transactivation inhibitory domain (TID)^10^ and residues located between the N-terminal transactivation and the DNA binding domains (Supplementary Fig. S1). This interaction forms a six-stranded anti-parallel β-sheet that blocks the tetramerization interface of the oligomerization domain, thereby keeping the protein in a dimeric state^11^. In addition, the N-terminal transactivation domain is also buried in this complex^9^ and therefore not available for interaction with transcriptional co-activators^12^. Inhibition, thus is based on two effects: inhibiting DNA binding and interaction with the transcriptional machinery, leading to a very effective overall inhibition, enabling oocytes to remain in this state for decades in humans.

Detection of DNA damage activates the stress kinases CHk1 (mainly through single strand breaks) and CHk2 (mainly through double strand breaks) which leads to the phosphorylation of S582^13,14^ located in a loop between the SAM domain^15,16^ and the C-terminal TID of TAp63α. While this modification itself does not influence the conformation of TAp63α, it recruits another kinase, CK1, which adds four more phosphate groups C-terminally to the CHk2 site^17^. Electrostatic repulsion between the phosphate groups and a stretch of aspartic acid residues results in the destabilization of the closed dimeric state and the formation of the open and active tetramer.

CK1 kinases belong to a group of enzymes that do not require phosphorylation in the activation loop for high activity and are therefore sometimes described as constitutively active^18^, only requiring a priming site on the substrate, for example provided by the phosphorylation of another kinase^19,20^ (although allosteric activators such as the DEAD-box RNA helicase DDX3 have been reported^21^ and also certain un-primed sequences can get phosphorylated by CK1^22–24^). In case of TAp63α, CHk2 acts as such a priming kinase. However, a situation in which CK1 only amplifies a CHk1/CHk2 signal would be highly dangerous for the long term survival of oocytes as the reproductive potential would be determined only by the activity of CHk1/CHk2. By measuring the phosphorylation kinetics of the four CK1 phosphorylation steps we could show that they follow different kinetic regimes^25^. While the first two phosphorylation events are very fast, the third one is ~ 20-fold slower. Interestingly, it is this third phosphorylation event that is necessary to produce the open state^17,25^ (the fourth is not necessary for the activation, only has a slight accelerating effect^25^). This slow kinetics of the decisive step serves as a safety measure. Activated, tetrameric TAp63α gets degraded fast, thus when the activation is slow because of a low level of DNA damage, the oocyte survives as activated TAp63α does not accumulate. Only when the damage level is high a corresponding fast activation would outcompete degradation of activated TAp63α, leading to cell death^25^. The trigger level for the activation of TAp63α, however, is species dependent. In humans the LD50 dose required for elimination of 50% of the ovarian reserve is between 2 and 20 Gy^26^, depending on the age of the patient, while in mice the LD50 value is 0.15 Gy. In guinea pigs, an LD50 value of 4 Gy was found and primordial oocytes of Rhesus monkeys are eliminated only after exposure to 70 –120 Gy^26,27^. Despite evolutionary divergence in radiation-sensitivity, TAp63α is highly conserved in mammals with only 9 amino acids difference between mouse and humans^6,17^, suggesting that additional safety mechanisms with different threshold levels exist in individual species. One such mechanism might be regulation through the C-terminal tail of CK1 kinases which have been shown to get auto-phosphorylated and inhibit the kinase activity^23,28–32^. Here we investigated the effect of auto-phosphorylation in the C-terminus of CK1δ on the phosphorylation kinetics of p63and show that auto-phosphorylation can almost completely inhibit the third, decisive phosphorylation step. Interaction between the kinase domain and the auto-phosphorylated C-terminus does not require a particular site but all phosphorylated serines seem to contribute. Important for a substrate to be able to compete with the inhibitory C-terminal domain of the kinase are hydrophobic residues following the phosphorylation site and that align with a nonpolar binding site on the kinase. We have chosen to investigate CK1δ as the best characterized kinase for TAp63α for which also co-crystal structures exist^25^. CK1ε and CK1α can phosphorylate TAp63α as well. As auto-inhibitory effects from the C-termini of these kinases have been reported^28–30,32^, similar effects as reported here can be expected for their phosphorylation kinetics of TAp63α. To better distinguish amino acids in the kinase from residues in the p63 phosphorylation activation domain (PAD) peptide we will use the three letter nomenclature for the kinase and single letters for the p63 peptide.

## Results

### Auto-phosphorylation of the C-terminal domain of CK1δ inhibits phosphorylation of S591

**Figure 1:**
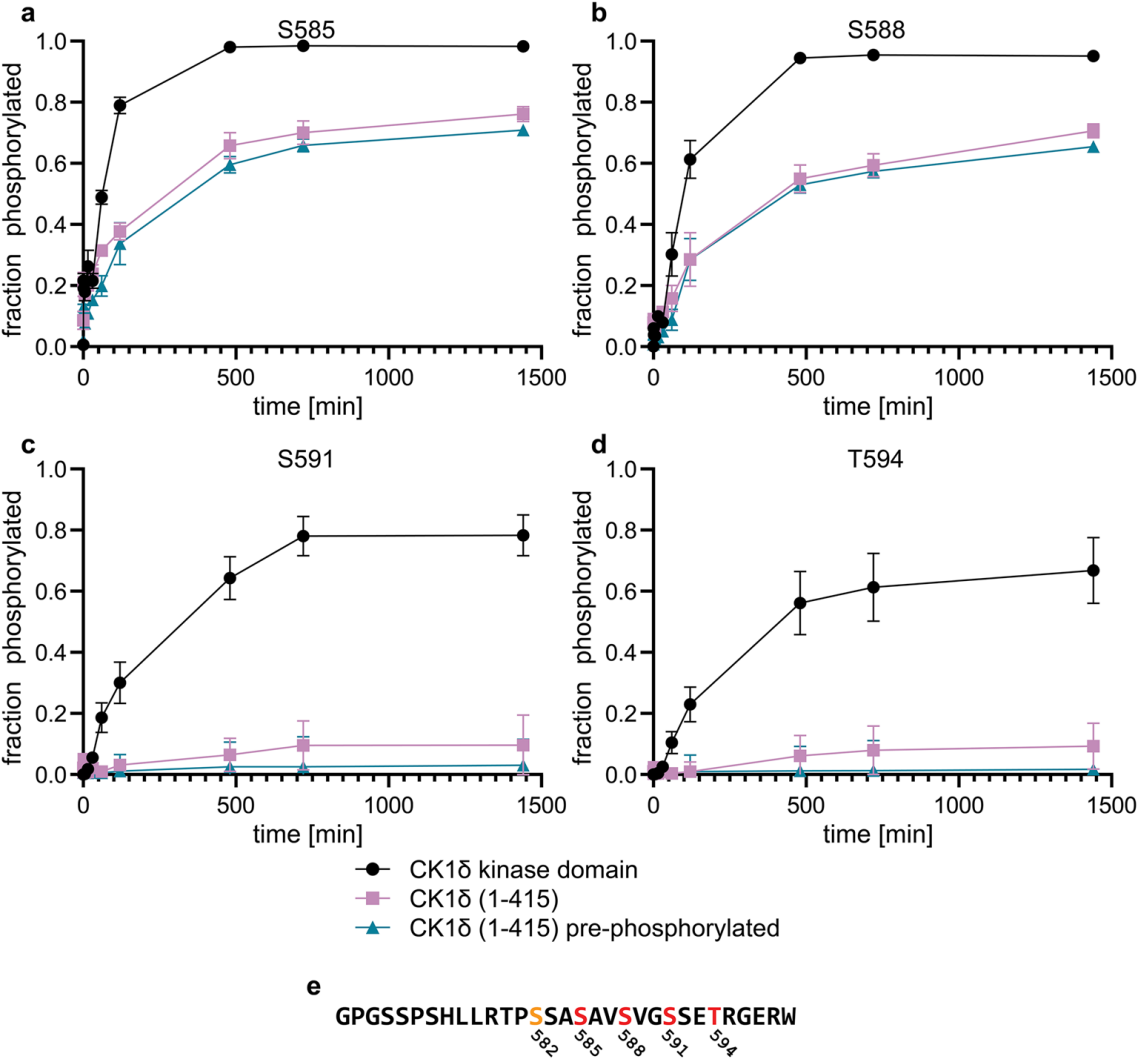
Auto inhibition is more predominant for the third phosphorylation site. (**a)** Phosphorylation kinetics on S585 in the p63 PAD peptide with the CK1δ kinase domain alone (black), full length kinase (red) and prephosphorylated full length kinase (blue) measured by mass spectrometry. The panels shown in (**b**–**d**) represent the phosphorylation kinetics on S588, S591 and T594 respectively. While the kinetics of S585 and S588 is slowed down, the phosphorylation of S591 and the subsequent T594 is almost completely inhibited by auto-phosphorylation in the C-terminus of full length kinase. (**e**) Sequence of the p63 PAD peptide. The CHK2 site is labeled orange, the four CK1 sites are marked red. Data points represent the average and standard deviation obtained from three individual experiments.

Phosphorylation of the kinase itself either in the kinase domain or in other domains can have a strong influence on the enzymatic activity^19^. For human CK1δ and its yeast homologues Hhp1 and Hhp2 it was found that phosphorylation of Thr220 located at the N-terminus of helix αG close to the substrate binding cleft results in changes in the phosphorylation kinetics^33^. While for most substrates the kinetics was slower in the Thr220 phosphorylated state, phosphorylation of S585 and S588 of TAp63α was surprisingly accelerated. We wanted to investigate the influence of further auto-phosphorylation events that mainly occur in the C-terminal domain of CK1δ to evaluate their potential influence on the activation of TAp63α in oocytes. We, therefore, mixed a p63 peptide containing all CK1 phosphorylation sites (G569 to R598) and primed on S582 with activated MK2 kinase^17^ with either the CK1δ isolated kinase domain or the full length kinase in a 1:2000 ratio (100 µM peptide concentration) and measured the phosphorylation kinetics by mass spectrometry. Here and in all following experiments we used a T586A mutant of the p63 PAD peptide. In our initial characterization of the phosphorylation kinetics we had seen that phosphorylation of T586 (which is not part of the CK1 consensus sequence) also occurs during the reaction at a slow rate. Mutating it does not change the kinetics of the four main phosphorylation sites but simplifies the analysis^25^. Figure 1 shows that the kinetics of the first two phosphorylation sites, S585 and S588, were slower with the full length kinase relative to the measurements with the isolated kinase domain. The kinetics for the decisive third phosphorylation event, S591, was even strongly inhibited, showing that the C-terminus has an inhibitory effect on the entire activation mechanism of TAp63α. Phosphorylation of the final fourth site, T594, was also strongly inhibited, mainly reflecting the sequential order that requires S591 to become phosphorylated first^25^.

Phosphorylation of the first two sites, S585 and S588, is very fast with the isolated kinase domain. To investigate if the only partial inhibition of their phosphorylation with the full length kinase is due to this very fast kinetics which occurs in parallel with the auto-phosphorylation of the C-terminus, we pre-incubated the full length CK1δ kinase with ATP for four hours before adding the p63 peptide. The phosphorylation kinetics of all four sites became only slightly slower, suggesting that the auto-phosphorylation is fast and no pre-incubation is necessary for an inhibitory effect in vitro (Fig. 1, Supplementary Fig. S2), consistent with a previous study that showed rapid auto-phosphorylation of CK1ε within five minutes^29^.

### The auto-phosphorylated C-terminus interacts with the kinase domain in a “fuzzy” mode

Auto-phosphorylation of the CK1δ C-terminus has previously been shown for Ser-318, Thr-323, Ser-328, Thr-329, Ser-331, and Thr-337^31,32^. To confirm that these sites play a role in inhibition of TAp63α activation in our in vitro assay, we mapped phosphorylation sites using mass spectrometry. These experiments resulted in the identification of full length kinase with a maximum of ten phosphorylated residues located at Thr323, Ser331, Thr344, Ser361, Ser382, Ser384, Thr397, Ser406, Ser407 and Ser411 following incubation with ATP for four hours (Supplementary Fig. S3). Phosphorylation of Thr220 in the kinase domain which was identified in a previous study^33^ was not detected in these experiments, which is caused by the very short peptide containing T220 resulting from trypsin digestion.

**Figure 2:**
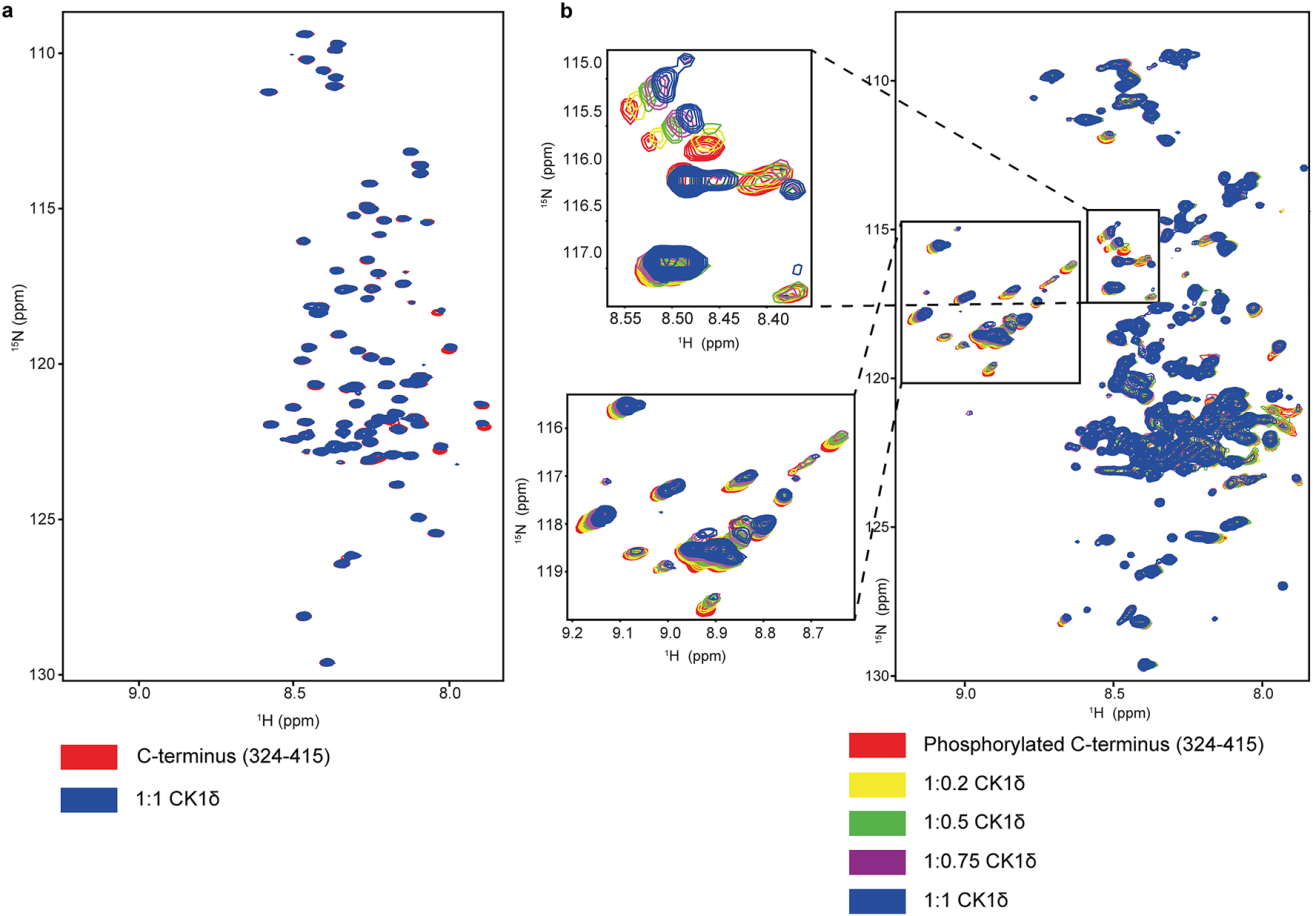
Interaction of the C-terminal domain of CK1δ with the isolated kinase domain. (**a**) Overlay of a [^15^N, ^1^H]-BEST-TROSY-HSQC of the ^15^N-labeled non-phosphorylated C-terminus (red) with a spectrum of the same sample after adding a 1:1 ratio of the unlabeled kinase domain (blue). (**b**) Overlay of [^15^N, ^1^H]-BEST-TROSY-HSQC spectra of a titration of the phosphorylated C-terminal domain (red) with increasing concentrations of the kinase domain. The signals of the phosphorylated residues are low field shifted relative to the unphosphorylated amino acids. The two inlets present regions of the spectrum showing interaction with the kinase domain.

We wanted to further characterize the interaction of the phosphorylated C-terminal domain with the kinase domain using NMR^25,34^. Titration of the unlabeled kinase domain to a sample of the ^15^N-labeld C-terminal domain (R324 to R415) resulted in only a few very minor chemical shift perturbations, suggesting that the interaction between the C-terminus and the kinase domain is very weak (Fig. 2a). Incubating the C-terminal tail with the kinase domain and ATP resulted in the appearance of strongly shifted resonances, characteristic for phosphorylated serine and threonine residues (Fig. 2b). In addition to ~ seven main signals the spectrum also contains several minor signals. This distribution shows that the phosphorylation level of the sites identified in the mass spectrometry analysis is not uniform and that auto-phosphorylation results in a mixture of many different states. Titration of this phosphorylated sample with the unlabeled kinase domain showed chemical shift differences for virtually all signals of phosphorylated residues and also for some additional resonances, demonstrating that the kinase domain has an increased affinity for phosphorylated peptides (Fig. 2b). The fact that chemical shift differences are seen for almost all phosphorylated residues further suggests that there is no single sequence that binds strongly and that is responsible for the inhibitory effect. Instead, all seem to contribute to interacting with the kinase domain, suggesting a “fuzzy” interaction mode as had been previously observed for other phosphorylated but intrinsically disordered proteins^35^. These titration experiments were carried out at a pH of 6.5 to reduce the chemical exchange of the amide protons with water and increase the signal intensity. This pH value, however, is close to the pKa of phosphoserine and phosphothreonine (5.6 and 5.9 respectively^36^). To ensure that the observed chemical shift differences are not due to slight pH changes during the titration which would influence the protonation state and thus also the chemical shift, we repeated the titration experiment at pH 7.5 (Supplementary Figs. S4, S5). The results show even stronger chemical shift differences for the phosphorylated residues, probably due to higher charge density of the phosphate groups at this pH. Increasing the salt concentration from 50 to 150 mM had almost no effect (Supplementary Fig. S6).

### Mutating T344, S361 and S370 have the strongest effect on relieving inhibition

**Figure 3:**
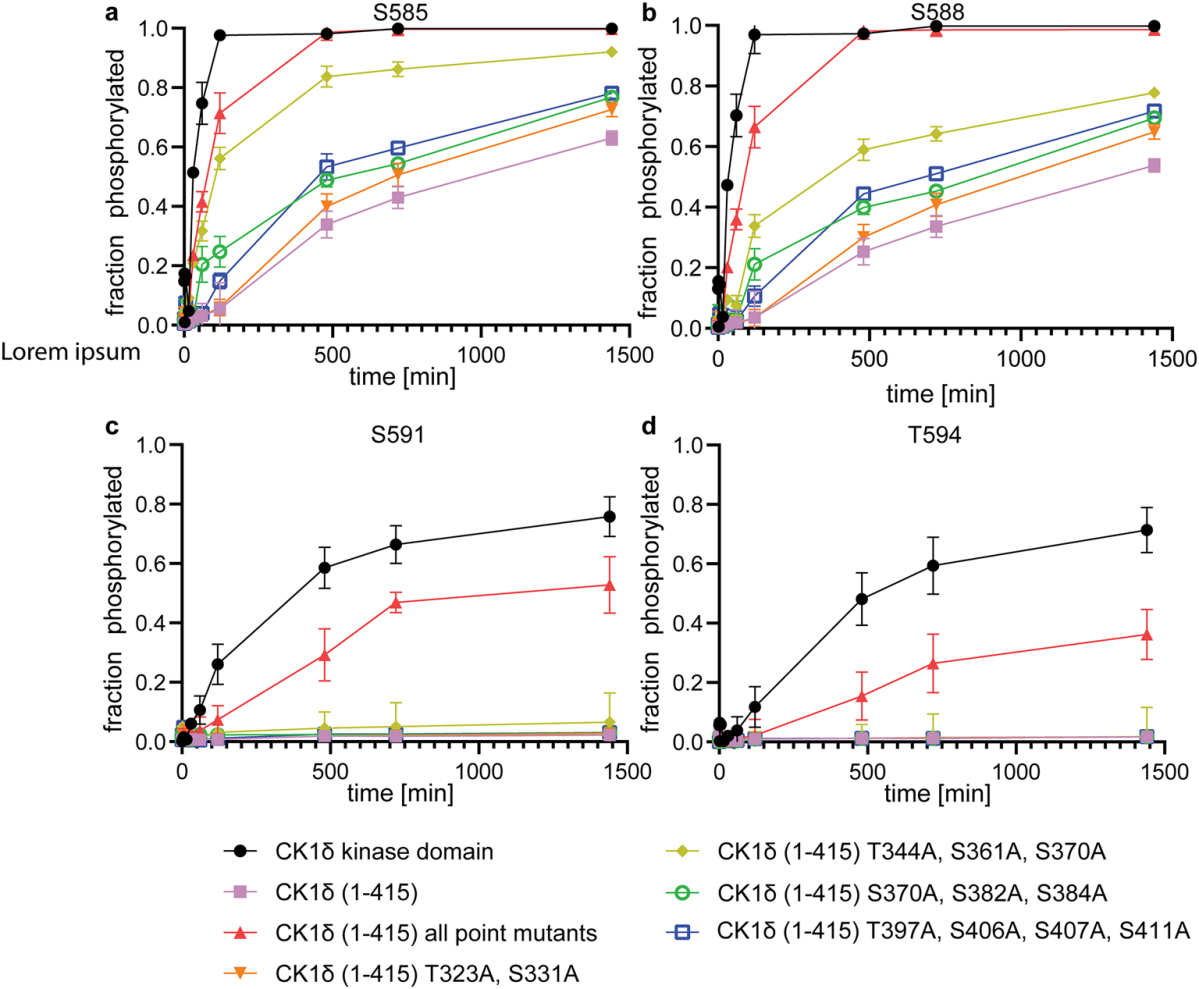
Mutations of phosphorylatable residues in the C-terminal domain of CK1δ cannot fully restore the phosphorylation kinetics. The phosphorylation kinetics were measured using mass spectrometry for S585 (**a**), S588 (**b**), S591 (**c**) and T594 (**d**). In each kinetic experiment a group of residues was mutated. The data show that not a single group can fully restore the kinetics and that all phosphorylation sites seem to contribute to the inhibitory effect. Phosphorylation of S591 can only be partially restored by mutating all serines that were found auto-phosphorylated by mass spectrometry. Data points represent the average and standard deviation obtained from three individual experiments.

We wanted to investigate this model of a fuzzy interaction of the phosphorylated C-terminal domain with the kinase domain further by mutational analysis. If all of the phosphorylated residues contribute, then the mutation of a single phosphorylation site should not restore full kinase activity. However, since in principle more than one site and any kind of combination of the identified ten phosphorylation sites might be particularly important, investigating all of them would result in an unrealistic number of mutants. To break down this task to a reasonable number we decided to mutate always groups of residues. In addition to the ten amino acids found via mass spectrometry we also included Ser370 into the mutation list. Ser370 was identified as a site phosphorylated by protein kinase A (PKA), protein kinase B (PKB), protein kinase Cα (PKCα), CDC-like kinase 2 (CLK2), and checkpoint kinase 1 (Chk1) in vitro and in vivo and its phosphorylation was shown to have a strong inhibitory effect^37–39^. In our mass spectrometry analysis Ser370 is located on a very small tryptic digestion fragment which was not detected (similar to the Thr220 containing peptide). As we cannot exclude that Ser370 also gets auto-phosphorylated we mutated it as well. Mutating the last four phosphorylation sites (Thr397, Ser406, Ser407, Ser411) to alanine resulted in an increase in the kinase activity relative to the wild type sequence but it was still significantly slower than the kinetics with the isolated kinase domain (Fig. 3a, b, Supplementary Fig. S7). Interestingly, mutating these residues had virtually no effect on the phosphorylation of the last two residues of the p63 PAD peptide (S591, T594) (Fig. 3c, d, Supplementary Fig. S7). Mutation of two other groups (Ser370, Ser382, Ser384 and Thr323, Ser331) had similar effects. A stronger effect was measured for mutating Thr344, Ser361 and Ser370, in particular on the phosphorylation kinetics of S585. A slight, but not significant increase was also seen for the phosphorylation of S591. Mutating all ten auto-phosphorylation sites and Ser370 resulted in almost wild type kinetics for the first two p63 phosphorylation sites (S585, S588) and in a partial restoration of the phosphorylation kinetics of S591 and T594. These data suggest that Thr344, Ser361 and Ser370 have the strongest inhibitory influence but even their mutation is not enough to effectively phosphorylate the decisive S591 residue which inhibits TAp63α activation. These data also demonstrate that the presence of even the fully mutated C-terminus has already an inhibitory effect on the phosphorylation kinetics in vitro. Similar results were obtained by repeating the experiment with selected mutants at an increased pH of 7.5 (Supplementary Figs. S8, S9).

### Hydrophobic residues surrounding the phosphorylation site are important for an effective competition with the inhibitory C-terminal domain

**Figure 4:**
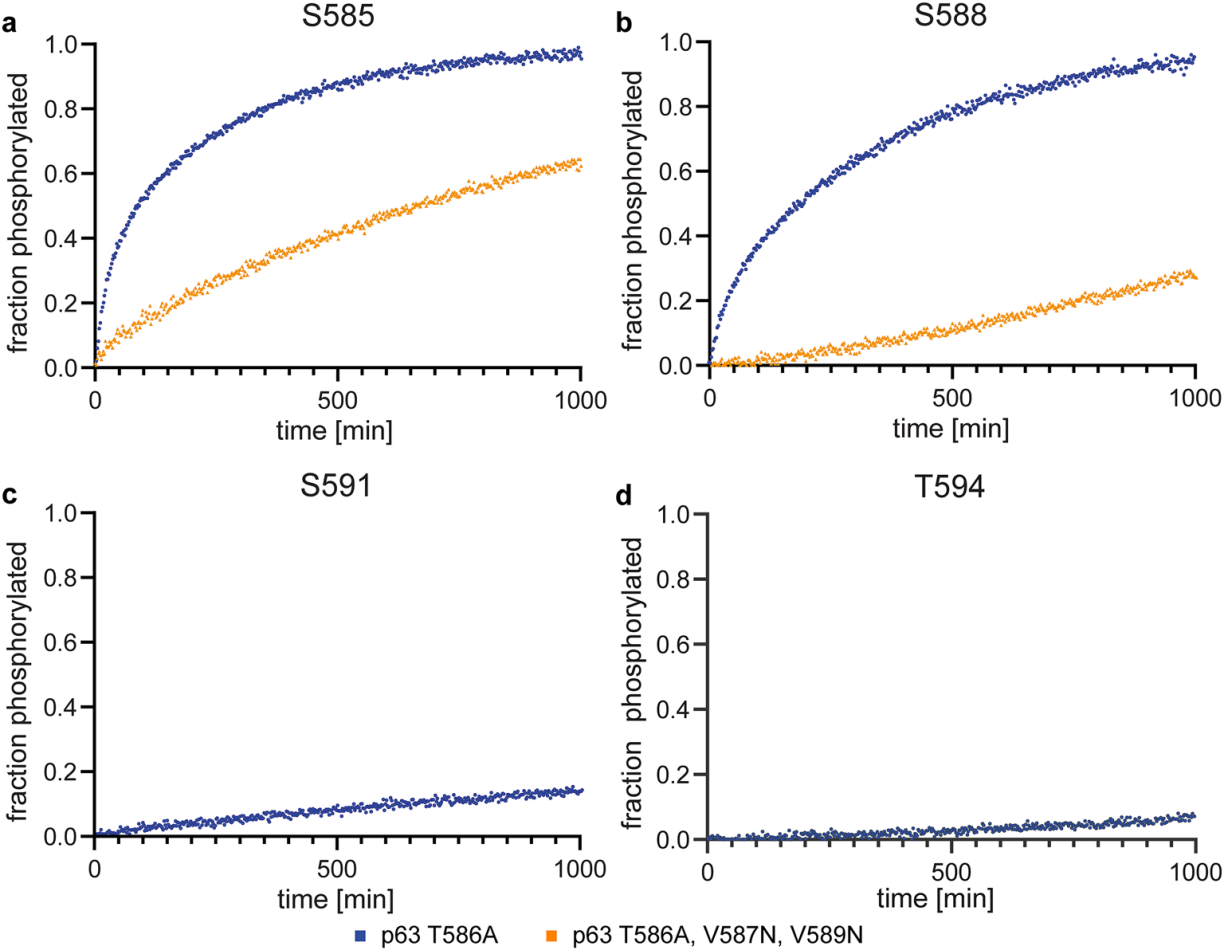
Comparison of the phosphorylation kinetics of a wild type p63 PAD peptide (except for the mentioned T586A mutation, shown in blue) with a mutated p63 PAD peptide (shown in yellow) measured with full length CK1δ. Mutating V587 and V589 of p63 PAD to asparagine as a small and polar but not phosphorylatable residue reduces the phosphorylation kinetics of S585 (**a**). The phosphorylation of S588 is even severely inhibited (**b**) and the phosphorylation of (**c**) S591 and (**d**) T594 can no longer be detected. Experiments with the V587N, V589N mutated PAD peptide were measured in triplicate, the data with the wild type PAD peptide in duplicate. The data shown represent one replicate.

The kinetic experiments so far have shown that the phosphorylation of S591 in the p63 PAD peptide is strongly inhibited by the auto-phosphorylation of CK1δ. This implicates that the first two phosphorylation sites (S585 and S588) are strong binding sites for CK1δ that can effectively compete with the phosphorylated C-terminal domain while S591 is a weak site. In a previous investigation we had shown that S592 and E593 are two key residues that determine the slow kinetics of the third site^25^. The residues C-terminally following each phosphorylation site interact with a small non-polar patch (centered around Leu173), helping to position the side chain of the serine or threonine residues in the active site. For S585, A586 (or the hydrophobic part of T586 in the wild type sequence) and the side chain of V587 provide this effect. For S588 the side chain of V589 acts as the hydrophobic anchor point. With S591 in the active site, however, S592 has to occupy this position which is less favorable than interaction of this site with the hydrophobic side chains following S585 and S588. The second important interaction is that of the side chain of E593 with a basic cluster in the CK1δ kinase domain (Arg127, Lys154, Lys171). This cluster has also been implicated in substrate selectivity for the different phosphorylation sites (degron and FASP peptides) in Per2^40^. The interaction of E593 with the basic cluster occurs when S588 is in the active site and also helps to anchor the peptide in the optimal position for S588 phosphorylation. Moving S591 into the active site requires that this interaction is broken. Mutating these two sites (S592V and E593G) indeed significantly accelerated the phosphorylation of S591, without much effect on the other sites^25^. The strongest effect was seen by mutating S592 to the hydrophobic residue valine, underlining the importance for a hydrophobic residue C-terminal to the phosphorylation site. To further investigate the importance of this previous result we mutated the two residues V587 and V589 to asparagine (as a small polar but not charged amino acid). These mutations indeed resulted in a slower phosphorylation kinetics of S585 and a strong inhibition of the phosphorylation of S588 with the full length CK1δ (Fig. 4). Phosphorylation of the next site, S591, was not detectable at all. Introducing the S592V and E593G mutations in addition, had only small effects on the kinetics for the first two sites but accelerated phosphorylation of S591 (Supplementary Figs. S10, S11). These data confirm the importance of the hydrophobic residue C-terminal to the phosphorylation site for effective competition with the inhibitory C-terminal domain of CK1δ.

### Kinetics of phosphorylation of PER2 is reduced by C-terminal auto-phosphorylation

**Figure 5:**
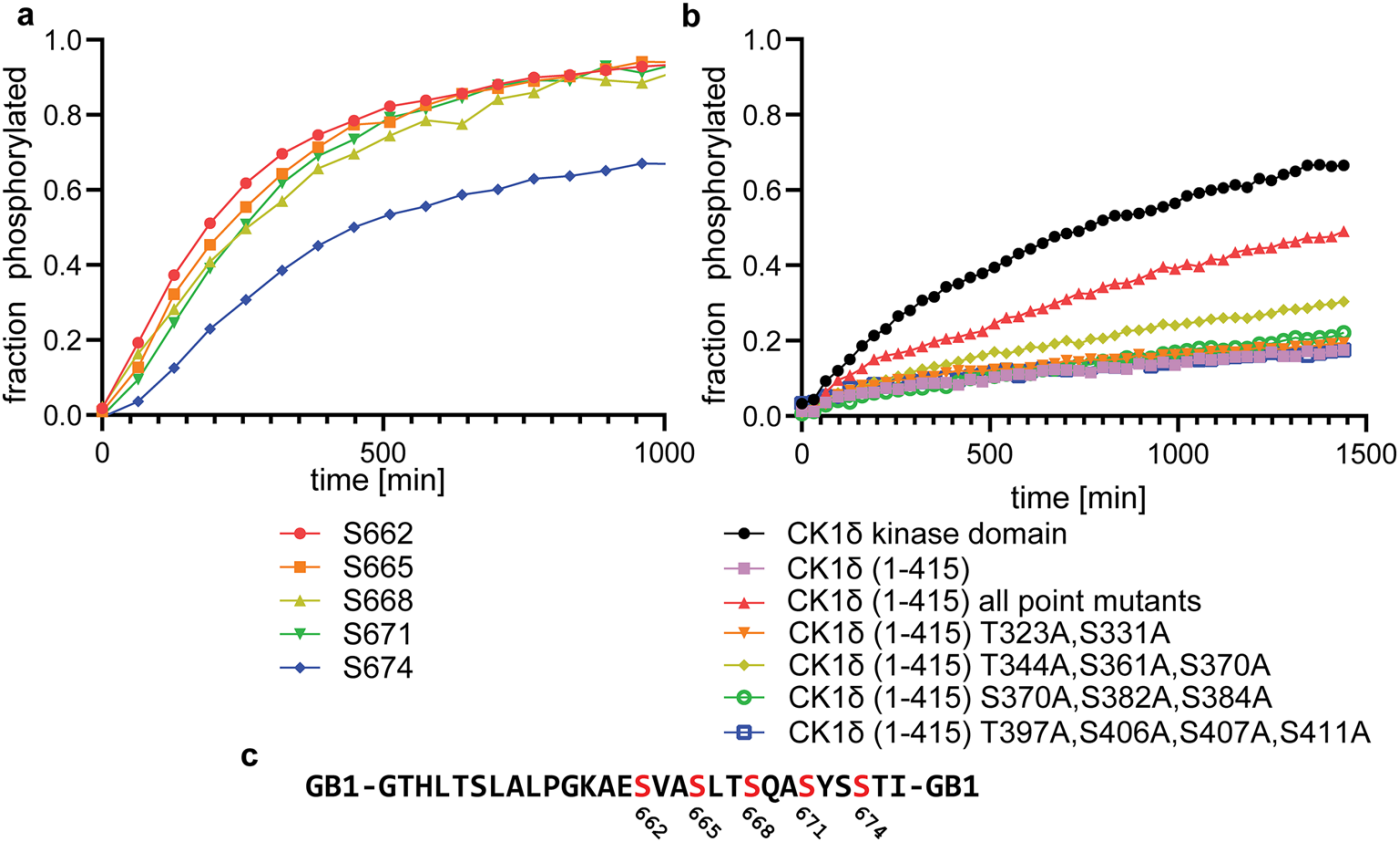
The phosphorylation kinetics of the human PER2 peptide is more uniform than the kinetics of the p63 PAD peptide. A C670A mutant was used to avoid problems with oxidation. (**a**) Phosphorylation kinetics of the five serines of the PER2 peptide with the CK1δ kinase domain in a 100:1 ratio. A duplicate with a 250:1 ratio was measured showing as expected slower but otherwise very similar kinetics (Supplementary Fig. S13). (**b**) Phosphorylation kinetics of S662 with different mutants of the CK1δ C-terminal domain. The same mutation groups as for the p63 PAD peptide experiments were used. Similar to the results with the p63 PAD peptide mutating the three residues Thr344A, Ser361A, Ser370A shows the strongest effect. Experiments were measured in duplicate, the data shown represent one replicate. (**c**) Sequence of the PER2 peptide, flanked by GB1 N- as well as C-terminally. The five phosphorylation sites are marked red.

CK1 kinases have also been shown to phosphorylate target proteins without a pre-phosphorylation event (in addition to their own C-termini), if certain sequence requirements are fulfilled. One such example is the PER2 protein which plays an essential role in the regulation of the circadian clock^23^. The relevant peptide stretch (T645 to I676, with a C670A mutation) contains five consecutive phosphorylation sites with a perfect CK1 spacing (+ 3). We wanted to investigate the influence of phosphorylation in the CK1δ C-terminus on the phosphorylation kinetics of PER2 as a member of this second class of targets (un-primed) that does not show such a big gap in the kinetics between different phosphorylation sites as TAp63α. To counterbalance the low solubility of this peptide which initially prevented the use of NMR spectroscopy to follow the phosphorylation kinetics we fused the small protein immunoglobulin G binding domain 1 of Streptococcal protein G (GB1) to its N- as well as its C-terminus. GB1 is often used as a solubility enhancer^41,42^ and this construct was soluble enough for our kinetic studies. After assignment of the GB1-PER2 peptide-GB1 construct using standard NMR experiments (Supplementary Fig. S12) we mixed the substrate with either the CK1δ kinase domain or full length CK1δ kinase in a 100:1 (Fig. 5a) or 250:1 (Supplementary Fig. S13) ratio and followed the reaction by NMR spectroscopy. This is a 10- to 20-fold higher kinase concentration compared to the experiments with the p63 PAD peptide and is necessary to measure the phosphorylation kinetics of this un-primed substrate. The overall phosphorylation of the PER2 sequence is significantly slower than the first two phosphorylation events of p63 (Fig. 5a) even at this elevated kinase concentration. The kinetics of the first four sites is quite similar without strong differences, reflecting the slow kinetics for priming the first residue, S662, and consecutive order of the following phosphorylation events as reported previously^23,40^. In contrast, the last phosphorylation event is significantly slower (Fig. 5a) which was independent of the substrate:kinase ratio (Supplementary Fig. S13). A similar, recent study of the phosphorylation of PER2 by CK1δ did not report differences between the first four and the last phosphorylation event^43^, suggesting that this difference in our measurement might be due to steric effects by the C-terminally following GB1 protein. To investigate the effect of phosphorylation in the C-terminus of CK1δ on the phosphorylation kinetics we repeated the measurements with the full length kinase. The results for the phosphorylation of S662 (Fig. 5b, Supplementary Fig. S14) as well as for S665, S668, S671 and S674 (Supplementary Figs. S15, S16) show a slower kinetics, similar to the effect measured for TAp63α. Using the same panel of mutants as for the p63 PAD peptide we measured again the strongest effect of relief of inhibition by mutating Thr344, Ser361 and Ser370 (Fig. 5b, c, Supplementary Figs. S14, S15, S16).

## Discussion

Members of the CK1, CK2 and GSK kinase families are not activated by phosphorylation in the activation loop and are often described as constitutively active^20,44,45^. Their predisposition for pre-phosphorylated sites enables them to add many phosphate groups in a row, thus amplifying the original phosphorylation signal. The requirement for such an amplification process is that phosphorylatable residues—serines or threonines—are located within a certain spacing. In case of CK1 kinases, this spacing is 3 residues in the direction of the C-terminus and in the case of the CK2 and GSK kinases three and four residues in the direction of the N-terminus, respectively^20,46^. The model that these kinases are not regulated at all has, however, changed with the characterization of the auto-phosphorylation as well as phosphorylation events by other kinases in the long, unstructured C-terminus^28–32^ as well as the observation of auto-phosphorylation within the kinase domain^33^. Inhibitory effects of C-terminal phosphorylations have been found for all members of the CK1 kinases, including CK1α^28,47^, CK1γ1-3^48^, CK1δ^23,31,32^ and CK1ε^29,30^. In addition to auto-inhibition, phosphorylation of the C-terminal tail of CK1δ was reported for the kinases PKA, Akt, CLK2, PKC and CHK1^37,38^. The distribution of the serine and threonine residues in the C-terminal domain do not follow a classical + 3 CK1 pattern. Still, previous data and the results reported here show that auto-phosphorylation of the C-terminus occurs fast and efficient. These data also suggest that all residues in the C-terminus of CK1δ that become phosphorylated contribute to auto-inhibitory effects. Our list of identified phosphorylated residues might not be complete and alternative methods to mass spectrometry might be necessary to obtain a complete overview of modified serines and threonines. For the interpretation of the data as a fuzzy interaction of the C-terminus with the kinase domain a completeness of the list is, however, not necessary.

A very recent study of the auto-inhibitory effect of the C-terminus of CK1δ comes to similar conclusions (https://doi.org/10.1101/2023.04.24.538174). However, this study found a predominant inhibitory effect for phosphorylation sites in the last 16 amino acids of the C-terminus. Interestingly, two splice forms of the CK1δ kinase differ by exactly these 16 amino acids^19^, suggesting that regulation of the activity of CK1δ could also be achieved by differential splicing.

The general mechanism of the autoinhibition of the kinase activity by the C-terminal domain does not seem to be that a specific phosphorylated sequence interacts strongly with the kinase domain, thereby blocking its enzymatic activity, but that of a fuzzy relatively weak interaction of many phosphorylated sites competing with substrates for access to the active site, similar to the interaction seen between the multiple-phosphorylated disordered protein Sic1 and Cdc4^35^. In CK1, the interaction of the phosphorylated C-terminus most likely occurs with two basic pockets located around Arg178, Lys224 and Arg127, Lys154, Lys171. In our crystal structures of differently phosphorylated p63 PAD peptides with the kinase domain of CK1δ (PDB accession codes 6RU6, 6RU7 and 6RU8), we have observed that the already phosphorylated sequence of the PAD peptide forms an α-helical structure and interacts via their phosphate groups with the basic pocket centered at Arg178 and Lys224^25^. The second basic pocket, located at Arg127, Lys154 and Lys171, interacts with the carboxyl group of E593 which contributes to the slow kinetics of the third phosphorylation site. Similar interactions of phosphorylated residues with the basic pockets were found in a recent crystal structure of the phosphorylated PER peptide in complex with the CK1δ kinase domain^43^.

For the activation of p63 the results shown here suggest that phosphorylation of the C-terminal tail of CK1 proteins by other kinases or by auto-phosphorylation has small effects on the kinetics of the first two sites. In contrast, the third site, which is the decisive one that leads to the opening of the closed dimeric state and the formation of the active tetramer is severely impacted. The first two sites are very good substrates for the CK1δ kinase. In contrast, our previous structure determination as well as Molecular Dynamics simulations have revealed that binding of the PAD peptide in a substrate mode with S591 in the active site is energetically less favorable as it would require interaction of a serine reside (S592) with the small hydrophobic patch of the kinase around Leu173 as well as breaking of the interaction between the side chain of E593 with the basic pocket of Arg127, Lys154, Lys171^25^. The peptide positioning for the phosphorylation of S591 thus constitutes a weak substrate with which the phosphorylated C-terminus of the kinase can compete effectively. The result is that the activation of TAp63α is further slowed down. Activated TAp63α gets degraded fast which is most likely a safety mechanism to avoid that spontaneous activation, low levels of kinase activity or low levels of DNA damage result in elimination of the oocyte.

The question remains how TAp63α can get effectively activated when auto-phosphorylation of the C-terminal domain of CK1δ effectively inhibits phosphorylation of S591. Previous investigations have shown that CK1ε and CK1δ in NIH 3T3 mouse fibroblasts are mostly unphosphorylated^32^. Treatment with calyculin A, a general phosphatase inhibitor, resulted in phosphorylation of both kinases. These results suggested that CK1δ is constantly de-phosphorylated by a phosphatase in the cellular environment to keep it active. If the C-terminal domain gets constantly de-phosphorylated as these data indicate, what role could it play in regulating the activity of the kinase in oocytes? In the case of CK1δ it was shown that S370 can get phosphorylated by the stress kinase CHK1. This kinase becomes activated upon DNA damage, in particular DNA single strand breaks. Phosphorylation of CK1δ by CHk1 could lead to partial inhibition of CK1 while at the same time phosphorylating TAp63α on S582, the priming site. As our results have shown that C-terminally phosphorylated CK1δ is still able to phosphorylate S585 and S588 but not S591 such a scenario would result in the built-up of S585 and S588 phosphorylated but still dimeric TAp63α. After de-phosphorylation of CK1δ, this pool of pre-activated TAp63α could then be effectively transformed into the active tetrameric state. Building-in such a delay could be an additional safety step for the oocyte to avoid cell death in case the damage is not severe as phosphatases might not only be important for increasing the activity of CK1 kinases but also for removing phosphorylation sites on pre-activated TAp63α before phosphorylation of S591^11^. A regulatory effect of a phosphatase, protein phosphatase 5, has indeed been shown for the regulation of the kinase activity of CK1ε as part of the circadian clock^49^. The role of CK1 kinases in oocytes, however, is complex and requires further investigations. In addition to CK1δ, CK1ε and CK1α can also phosphorylate TAp63α, although inhibitor studies have suggested that CK1ε plays a less important role^17^. CK1 isoforms, in particular CK1α, are also involved in regulation of DSB repair in oocytes^50^ and effective DSB repair would prevent oocyte death. Thus the level of DNA damage that results in oocyte death that is encoded in the activation kinetics of TAp63α could be fine-tuned by several kinases (CHK1/CHK2 and different CK1 enzymes). The results reported here suggest that in addition to kinases, phosphatases might also represent important regulatory elements and could contribute to the different sensitivities of oocytes toward DNA damage observed in different species.

## Methods

### Protein expression, purification, and phosphorylation

Recombinant human CK1δ (gift from the laboratory of Stefan Knapp) was subcloned into the pMAL-c4x vector (architecture of the construct:

MBPtag—TEVsite—Full length CK1δ—Strep-Tag). The protein, bearing a C-terminal Strep-tag, was co-expressed with lambda phosphatase in BL-21(DE3)-R3-Rosetta (SGC Oxford) in 2xYT medium. Protein expression was induced with 1 mM IPTG and expression was carried out for 18 h at 16 °C. Cells were harvested by centrifugation, resuspended in Buffer A (25 mM Hepes pH 7.8, 0.5 M NaCl, 20 mM β-mercaptoethanol) supplemented with RNAse (Sigma), DNAse (Sigma), protease inhibitor cocktail (homemade) and lysed by sonication. The lysate was cleared by centrifugation at 4 °C. The supernatant was passed through an equilibrated dextrose column, washed with 5 column volumes (CV) of buffer A and eluted with 20 mM Maltose in Buffer A. Afterwards, the MBP tag was cleaved using TEV protease at a 1:50 (w/w) ratio of TEV to protein over night at 4 °C. The protein was loaded onto a Strep column (StrepTrap HP, Cytiva) and eluted using 2 mM desthiobiotin (Sigma) and finally purified by size exclusion chromatography, SEC (HiLoad 16/600 Superdex 200, Cytiva) in kinase NMR buffer (50 mM Bis–Tris pH 6.5, 50 mM NaCl, 10 mM MgCl_2_, 0.5 mM TCEP).

Phosphorylation of full length CK1δ for MS–MS analysis and kinetics measurement was done by incubating the protein at 298 K with 10 mM ATP (Sigma) for 4 h and quenching the reaction with 20 mM EDTA. The phosphorylated protein was purified using SEC (Superdex 75 10/300 column, Cytiva) in kinase NMR buffer.

The human PER2(645–676) peptide was expressed as a C670A mutant, fused at its C- as well as its N-terminus with the immunoglobulin G binding domain 1 of Streptococcal protein G (architecture of the construct: GB1 HIS_8_—TEVsite—hPER2—GB1 Strep-tag) in a pET16b expression vector and was isotopically labeled by expression in M9 minimal media for 18 h at 20 °C after induction with 0.8 mM IPTG at an OD of 0.6. After resuspension in Buffer A (50 mM Tris pH 7.8, 0.5 M NaCl, 20 mM imidazole) and sonication the lysate was loaded on an immobilized metal affinity chromatography (IMAC) column (HiTrap IMAC Sepharose FF, Cytiva), washed with 10 CV of Buffer A and eluted with 300 mM imidazole in Buffer A. Binding and elution from a Strep column eliminated truncated constructs and the protein was finally purified by SEC (HiLoad 16/600 Superdex 75, Cytiva) in NMR Kinase buffer. Phosphorylation was done by incubating the protein at 298 K with 10 mM ATP (sigma) with a 1:100 ratio of kinase to protein for 24 h. The reaction was quenched by addition of 20 mM EDTA. The phosphorylated protein was purified by SEC (Superdex 75 10/300 column, Cytiva) into kinase NMR buffer. The phosphorylation state was confirmed by Mass Spectrometry. The sample was used for the assignment of the peptide.

The C-terminal domain of CK1δ (GFP-His_6_-3Csite-C-terminal domain-His_6_) was isotopically labeled by expression in M9 medium for 16 h at 22 °C following induction with 500 µM IPTG at an OD of 0.6. The purification protocol followed the one established for the hPER2 peptide (buffer A: 25 mM Tris pH 8.0, 200 mM NaCl, 30 mM imidazole; washing with 10 CV; elution with buffer B: 25 mM Tris pH 8.0, 200 mM NaCl, 400 mM imidazole). The expression tag and the peptide were cleaved by incubation with 3C protease overnight at 4 °C along with dialysis to reduce the NaCl and imidazole concentrations to 50 mM. Cation exchange chromatography was performed to eliminate GFP and the protease. The protein was subjected to SEC in kinase NMR buffer (Superdex 75 10/300 GL, GE Healthcare). Phosphorylation was done incubating the protein at 298 K with 10 mM ATP (Sigma) with (1:100) ratio of kinase to protein for 48 h. Quenching of the reaction was done with EDTA. The phosphorylated protein was purified using SEC (Superdex 75 10/300 column, Cytiva) in kinase NMR buffer. Furthermore, the phosphorylation state was confirmed by Mass Spectrometry.

Purification and activation of MK2Δ1-41 were performed as described previously^17^.

All p63 peptide purification and activation was done similar as reported earlier^17^.

### NMR spectroscopy

Samples for NMR experiments were prepared in kinase NMR buffer (50 mM Bis–Tris pH 6.5, 50 mM NaCl, 10 mM MgCl_2_, 0.5 mM TCEP). Experiments were measured at a sample temperature of 298 K. Assignments of GB1-hPER2-GB1 and the C-terminal domain of CK1δ at different phosphorylation states were performed using constant-time HNCACB, HN(CO)CACB and HN(CA)CO experiments.

Phosphorylation kinetics of p63 were recorded using sample volumes of 180 µl placed in 3-mm capillaries and final peptide concentrations of 250 µM. The kinase concentration was 125 nM (1:2000). The phosphorylation kinetics of GB1-hPER2-GB1 were measured in standard NMR tubes of 5 mm with a volume of 600 µl using 150 µM of PER2 and 600 nM (1:250) or 1.5 µM (1:100) kinase. All NMR experiments with peptides were performed at least twice under identical conditions. The general sample composition of a kinetic sample was as follows:^15^N-labeled peptide (250 µM), protease inhibitor (1×), phosphatase inhibitor (1×), ATP (10 mM), CK1 kinase (125 nM), buffer (to 200 µl).

A series of [^15^N, ^1^H]-BEST-TROSY correlation spectra were recorded throughout the kinetic reaction with NMR instruments operating at ^1^H frequencies ranging from 600 to 950 MHz.

### MALDI-MS based enzymatic assay (data shown in Fig. 1)

Kinetics were measured similar as reported before^17^. Full length kinase and pre-phosphorylated full length kinase were passed through SEC column on the day of the experiment to avoid soluble aggregates. The reaction mixture contained 100 µM p63 PAD peptide, prephosphorylated at S582, 50 nM kinase, 10 mM ATP, 1× Protease inhibitor, 0.5 mM TCEP in NMR Buffer. Kinetics were measured at 298 K. Aliquots of these reactions were quenched by the addition of 0.1% TFA at certain time points.

### ESI–MS based enzymatic assay (data shown in Fig. 3)

All the kinase was subjected to SEC prior to the kinetics on the same day at 50 nM concentration in the same buffer as described for the NMR experiments. The reaction mixture contained 100 µM p63 PAD peptide, prephosphorylated at S582, 50 nM kinase, 10 mM ATP, 1 × Protease inhibitor, 0.5 mM TCEP in NMR Buffer. Kinetics were measured at 298 K. Aliquots of these reactions were quenched by the addition of 0.1% TFA at certain time points. All the reactions were carried out in triplicates. The samples were measured on a LC-ESI-TOF (Agilent 6230B) Mass Spectrometry instrument. An external standard was used to correct for the deviating behavior of differentially phosphorylated peptides^17^.

### Sample preparation for mass spectrometry for phosphorylation-site identification

The purified CK1δ protein was reduced with 10 mM TCEP and alkylated with 40 mM chloroacetamide in 50 mM Tris–HCL and NaCl buffer. 6 µg of the protein were subjected to enzymatic digestion using Trypsin (Promega, V5113) at a weight-to-weight ratio of 1:100 (Trypsin:Protein). The digestion was carried out overnight at 37 °C in a final Urea concentration of 1 M. The peptides were cleaned up with Empore C18 stage tipping and dried right away for shooting.

### Mass spectrometry (LC-MS^2^)

Dried peptides of the sample were resuspended in 2% (v/v) acetonitrile/1% (v/v) formic acid solution and shot similar to earlier publication^51^. Simply, peptides were separated with Easy-nLC 1200 (Thermo Fisher Scientific) and kept at 50 °C using an integrated column oven. Individual peptides were eluted by a nonlinear gradient from 4 to 32% acetonitrile over 50 min, followed by a step-wise increase to 76% acetonitrile in 6 min, which was kept for another 9 min and sprayed into a QExactive HF massspectrometer (Thermo Fisher Scientific). Full-scan MS spectra (300–1650 m/z) were acquired with a resolution of 60,000 at m/z 200, maximum injection time of 20 ms and AGC target value of 3 × 10^6^.

The 10 most intense precursors were selected for fragmentation (Top 10) and isolated with a quadrupole isolation window of 1.4Th. MS2 scans were acquired in centroid mode with a resolution of 30,000 at m/z 200, a maximum injection time of 54 ms, AGC target value of 1 × 10^5^. Ions were then fragmented using higher energy collisional dissociation (HCD) with a normalized collision energy (NCE) of 27; and the dynamic exclusion was set to 20 s to minimize the acquisition of fragment spectra of already acquired precursors.

### Proteomics data analysis

Raw data was analyzed with Proteome Discoverer 2.4 (ThermoFisher Scientific). SequenceHT node was selected for database searches of MS2-spectra. Human trypsin digested proteome (Homo sapiens SwissProt database (TaxID:9606, version 12 March 2020)) was used for protein identifications. Contaminants (MaxQuant “contamination.fasta”) were determined for quality control. Carbamidomethyl (C, + 57.021) at cysteine residues were set as fixed modifications. Phosphorylation (+ 79.966 Da) on Ser, Thr, and Tyr residues, methionine oxidation (M, + 15.995) and acetylation (+ 42.011) at the protein N-terminus were set for dynamic modifications. Precursor mass tolerance was set to 7 ppm and fragment mass tolerance was set to 0.5 Da. Default percolator settings in PD were used to filter perfect spectrum matches (PSMs). Reporter ion quantification was achieved with default settings in consensus workflow. The mass spectrometry proteomics data have been deposited to the ProteomeXchange Consortium via the PRIDE^52^ partner repository with the dataset identifier PXD043233.

### Supplementary Information

Supplementary Figures.

## Data Availability

The mass spectrometry datasets generated during the current study are available in the ProteomeXchange Consortium via the PRIDE partner repository, dataset identifier PXD043233.

## Abbreviations

CHK2: Checkpoint kinase 2
CHK1: Checkpoint kinase 1
CK1: Casein kinase 1
TID: Transactivation inhibitory domain
