# Supplementary material for: Fuzzy interactions between the auto-phosphorylated C-terminus and the kinase domain of CK1δ inhibits activation of TAp63α
Source: Sci Rep. 2023 Sep 30;13:16423. doi: 10.1038/s41598-023-43515-x (PMC10542812; doi:10.1038/s41598-023-43515-x)
Supplement: Supplementary file 1 — Supplementary Figures. [file 41598_2023_43515_MOESM1_ESM.docx]

**Supplementary Figures**


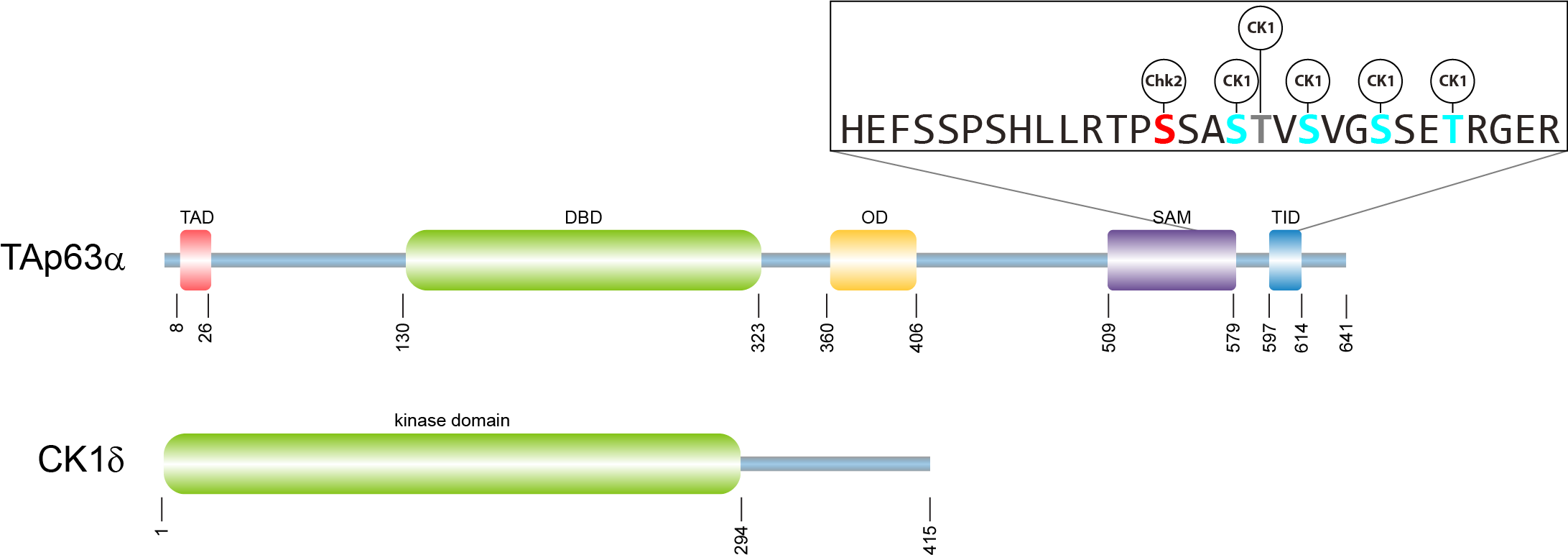


**Figure S1.** Domain structure of TAp63α (top) and CK1δ (bottom). Amino acid numbers of the beginning and the end of the domains are indicated. TAD: transactivation domain, DBD: DNA binding domain, OD: oligomerization domain, SAM: sterile alpha motif domain, TID: transactivation inhibitory domain. For TAp63α the phosphorylation sites that are relevant for the activation are indicated. S582 (shown in red) is the priming site, phosphorylated by CHK2. All CK1 sites (S585, S588, S591 and T594) are shown in blue. T586 is a non-canonical phosphorylation site that gets phosphorylated in full length TAp63α with a slow kinetic as well. In our experiments described in this manuscript we had mutated T586 to alanine because this mutation does not change the kinetic, is not relevant for the activation mechanism but simplifies the evaluation of the NMR and in particular the mass spectrometry data.


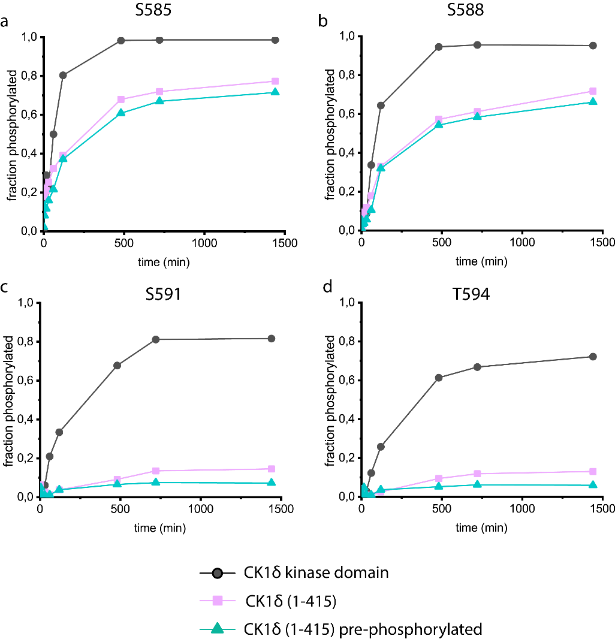


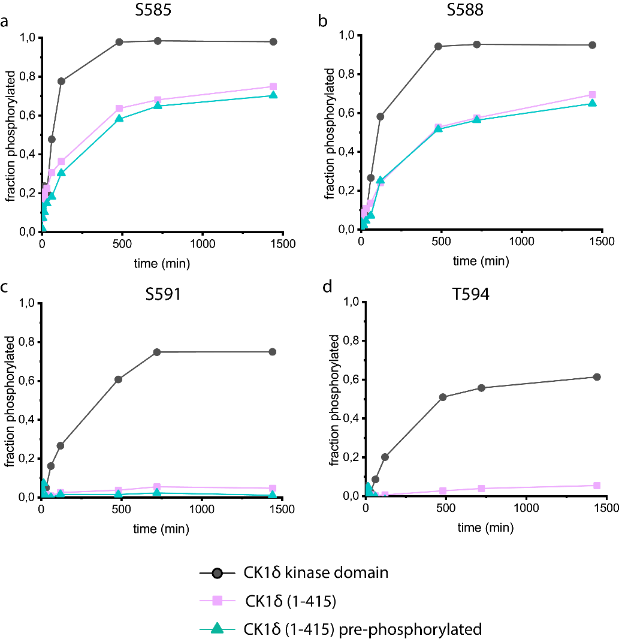


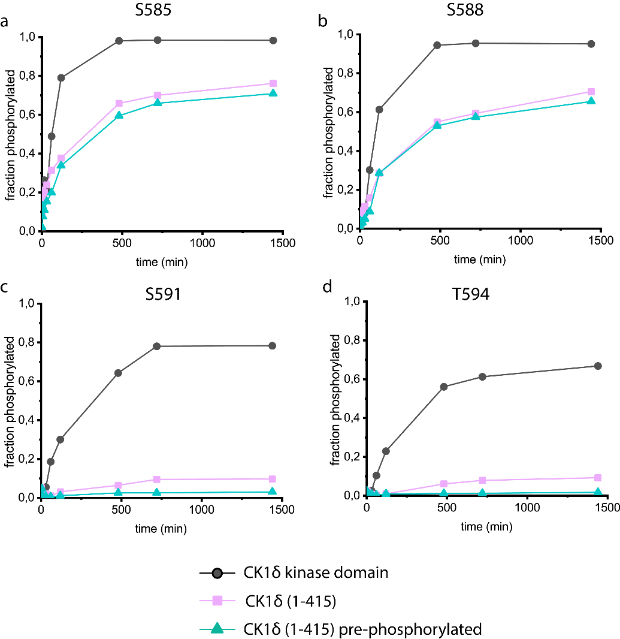


**Figure S2.** Replicates of the data shown in Figure 1.

**10 20 30 40 50 60
MELRVGNRYR LGRKIGSGSF GDIYLGTDIA AGEEVAIKLE CVKTKHPQLH IESKIYKMMQ

 70 80 90 100 110 120
GGVGIPTIRW CGAEGDYNVM VMELLGPSLE DLFNFCSRKF SLKTVLLLAD QMISRIEYIH

 130 140 150 160 170 180
SKNFIHRDVK PDNFLMGLGK KGNLVYIIDF GLAKKYRDAR THQHIPYREN KNLTGTARYA

 190 200 210 220 230 240
SINTHLGIEQ SRRDDLESLG YVLMYFNLGS LPWQGLKAAT KRQKYERISE KKMSTPIEVL

 250 260 270 280 290 300
CKGYPSEFAT YLNFCRSLRF DDKPDYSYLR QLFRNLFHRQ GFSYDYVFDW NMLKFGASRA

 310 320 330 340 350 360
ADDAERERRD REERLRHSRN PATRGLPSTA SGRLRGTQEV APPTPLTPTS HTANTSPRPV

 370 380 390 400 410
SGMERERKVS MRLHRGAPVN ISSSDLTGRQ DTSRMSTSQI PGRVASSGLQ SVVHR**


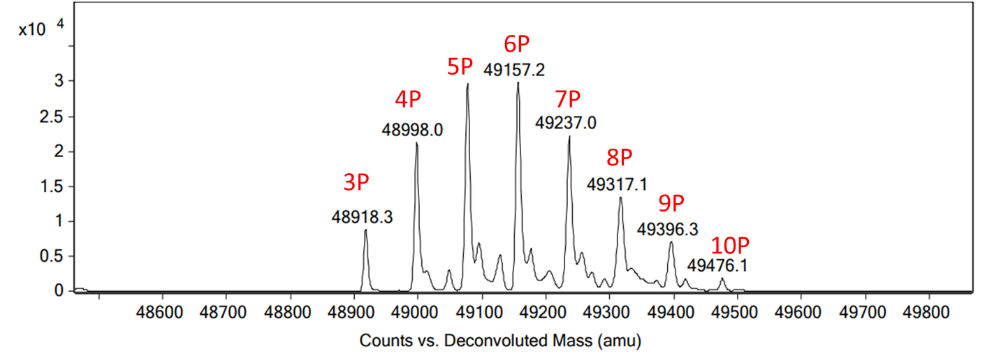


**Figure S3.** All phosphorylated serine and threonine identified after 4hrs of incubation of full length CK1δ kinase with ATP followed by trypsin digestion and LC-MS/MS analysis are labeled in red. All sequences that were detected in the mass spectrometry experiment are shown in green. Ser370 is marked in addition, although it could not be detected due to the small size of the tryptic digestion peptide it is located in. A maximum of ten phosphorylated sites was found.


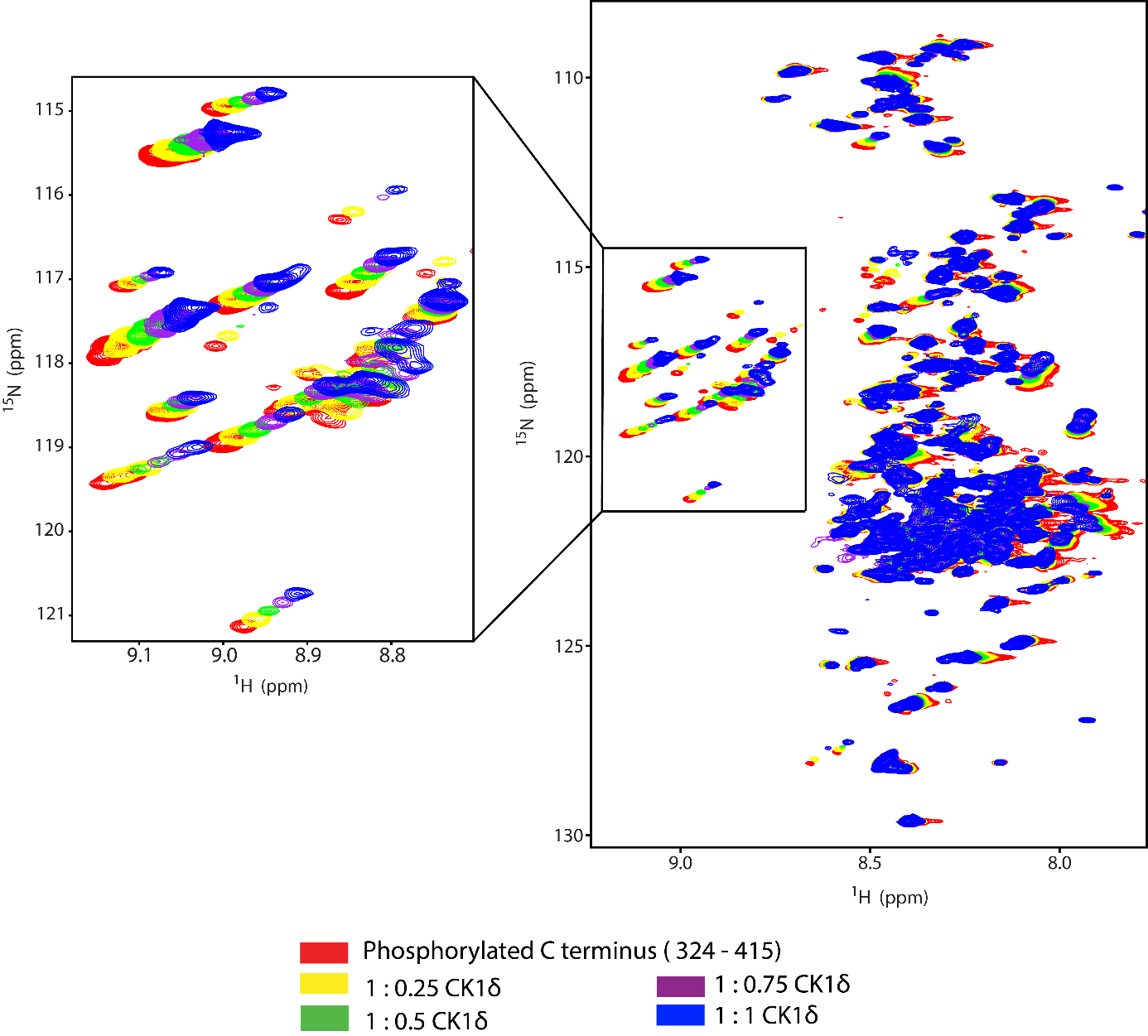


**Figure S4.** Titration experiment of the phosphorylated C-terminus of CK1 with the kinase domain at pH 7.5 and 50 mM NaCl. The shifts are larger than the shifts observed at pH 6.5 probably due to the higher charge density of the phosphate groups at this pH.


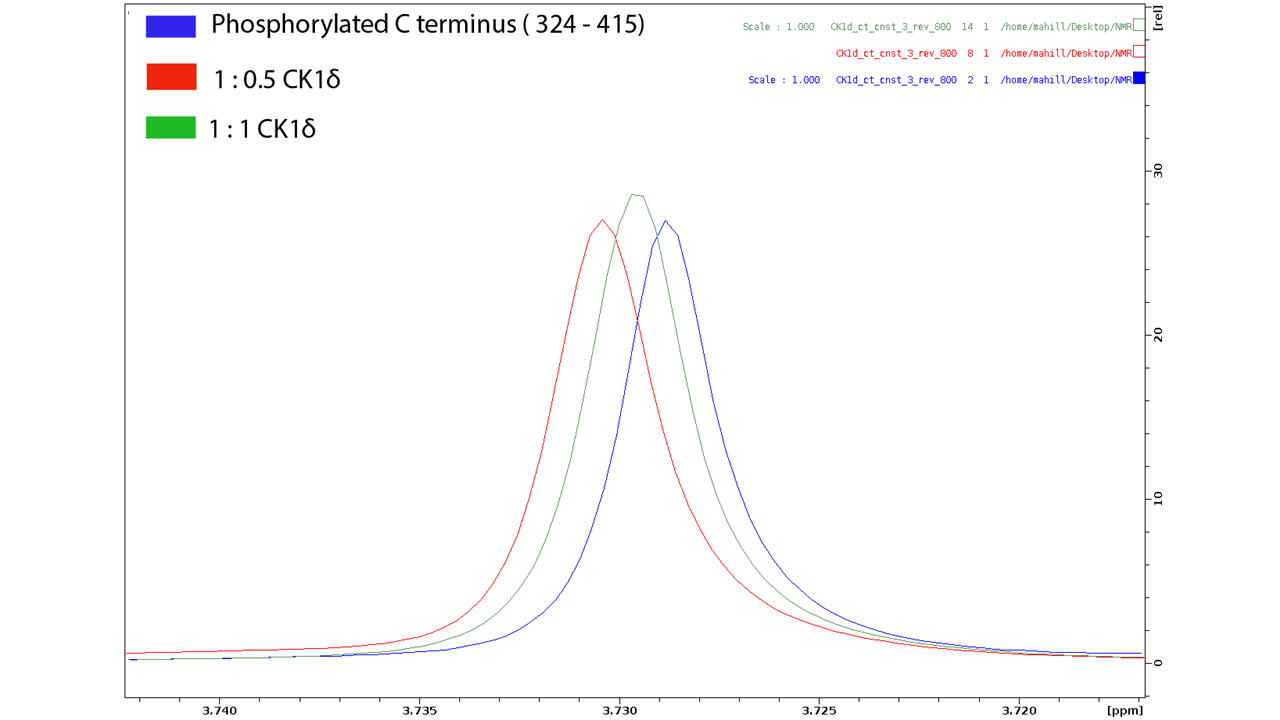


**Figure S5.** Position of the Tris buffer signal during the titration experiment of the phosphorylated C-terminus with the kinase domain. The peak positions for the titration points 0%, 50% and 100% are shown and reveal only very small shift differences, indicating a stable pH value during the experiment.


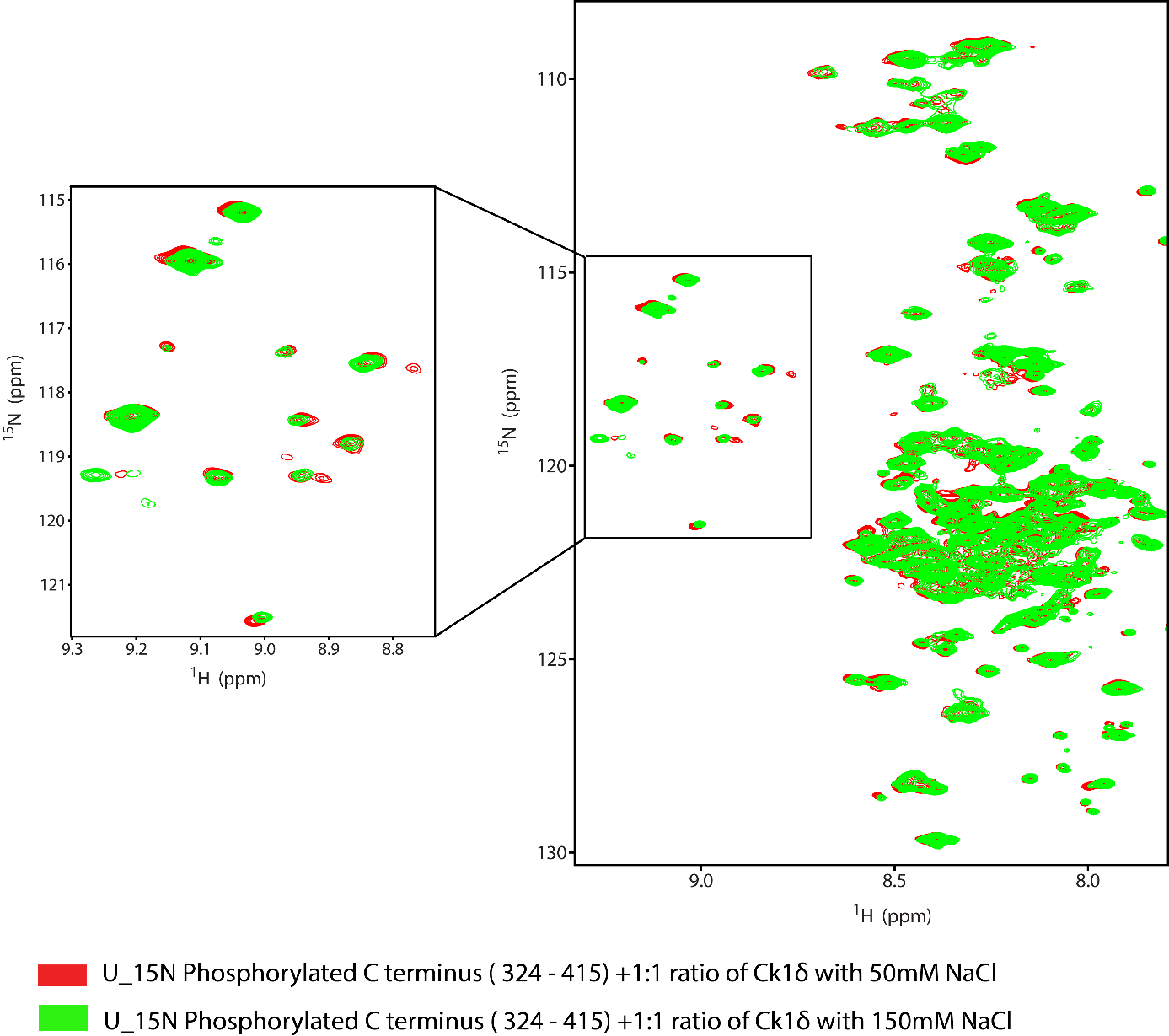


**Figure S6.** Investigating the effect of salt concentration. To the sample representing a 1:1 ratio of phosphorylated C-terminus and kinase domain at 50 mM NaCl (red spectrum) NaCl in Tris buffer, pH 7.5 was added to a total concentration of 150 mM (green spectrum). Only very small shifts in peak position for some residues are visible indicating that the salt concentration does not have a major influence on the observed interaction. A small amount of degradation is also visible from additional peaks.


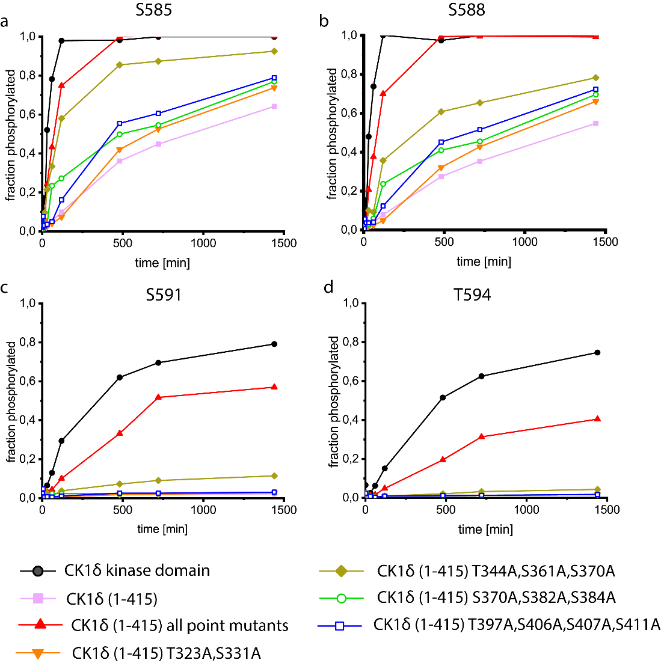


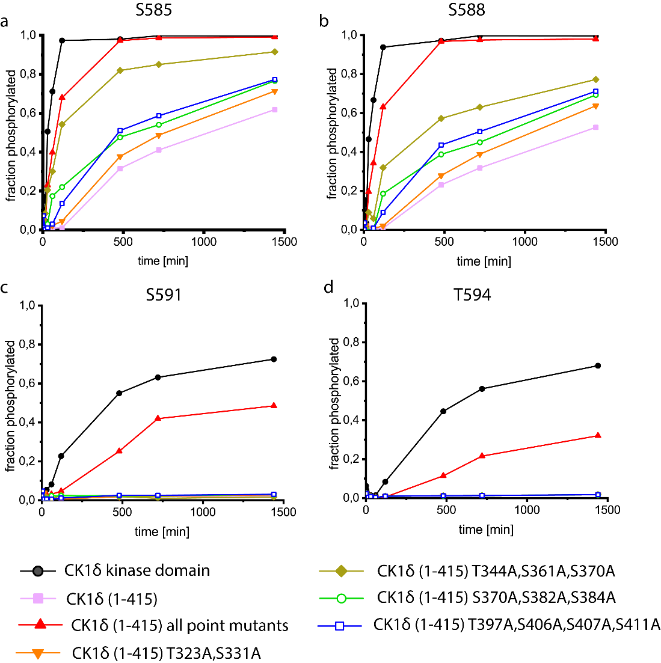


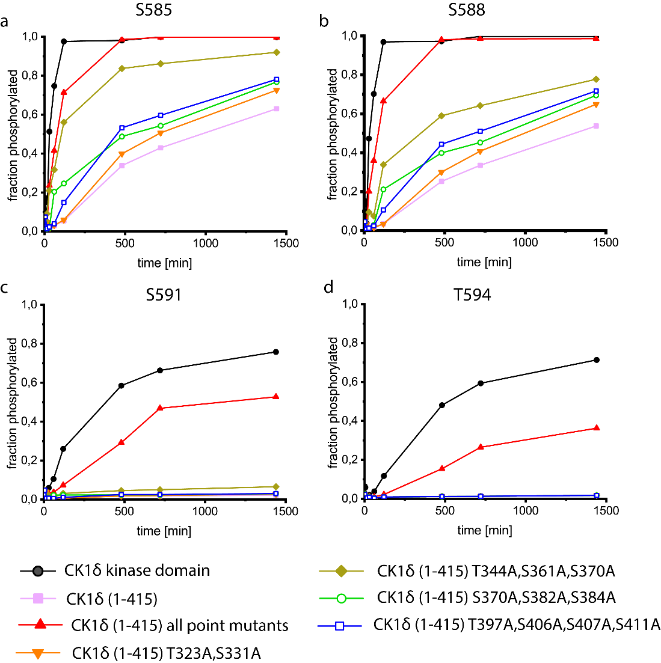


**Figure S7.** Replicates of the data shown in Figure 3.


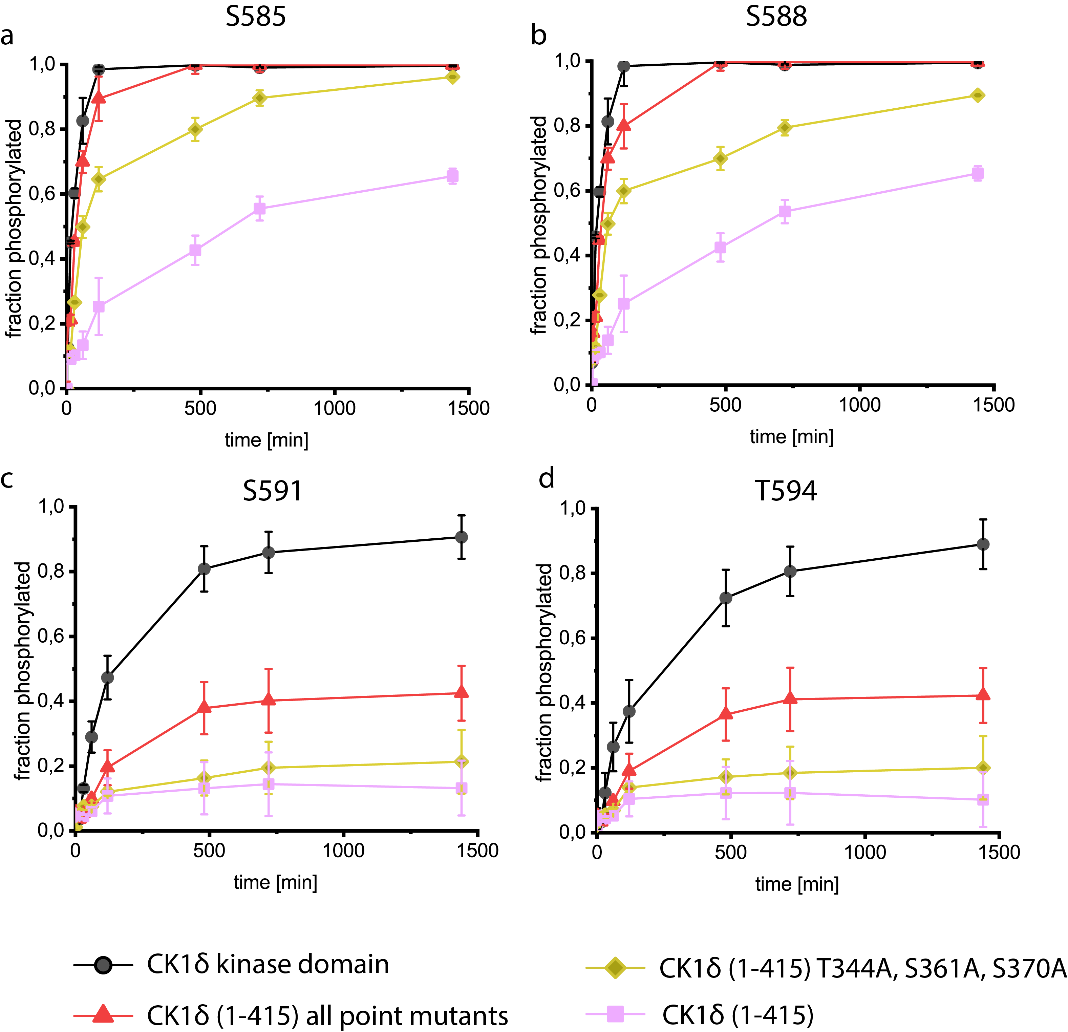


**Figure S8.** The mass spectrometry based measurement of the phosphorylation kinetics reported in Figure 3 was repeated at pH 7.5 and 150 mM NaCl for some selected mutants. The results are similar to the measurements at pH 6.5.


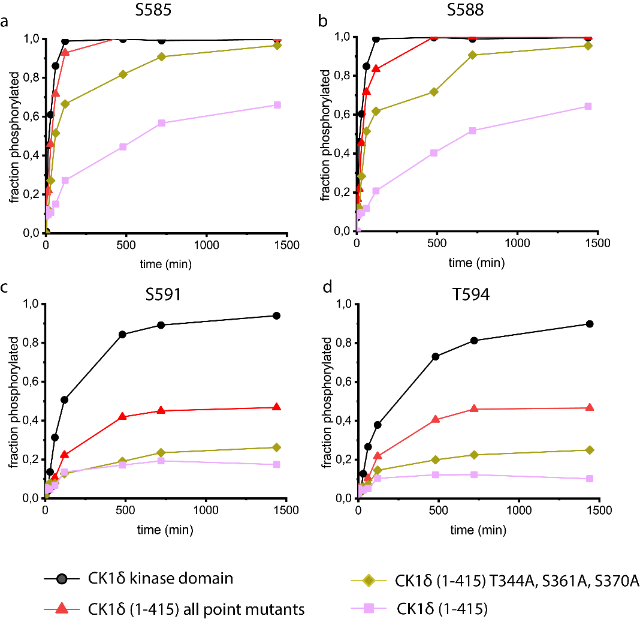

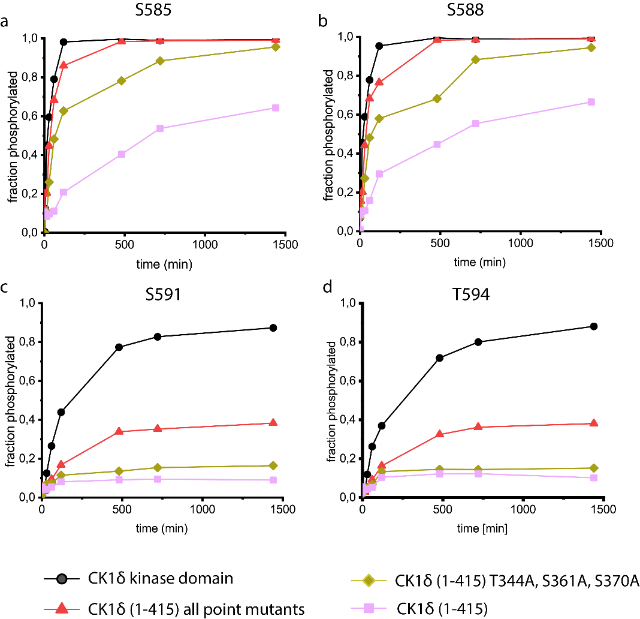


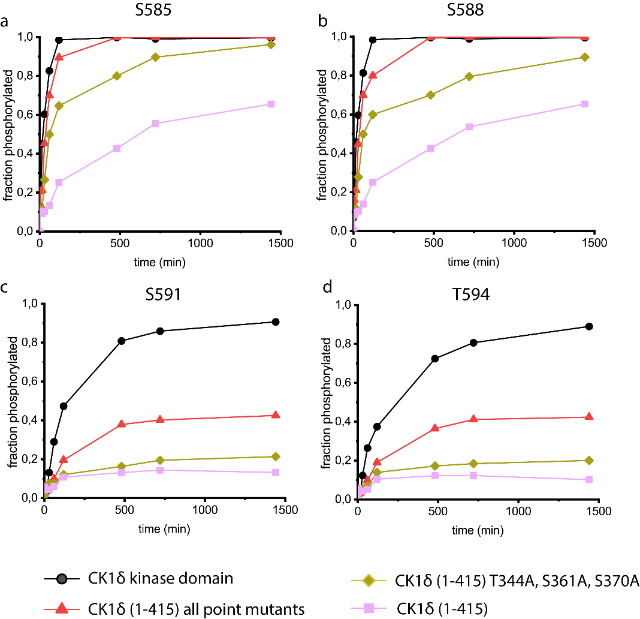


**Figure S9.** Replicates of the data shown in Figure S8.


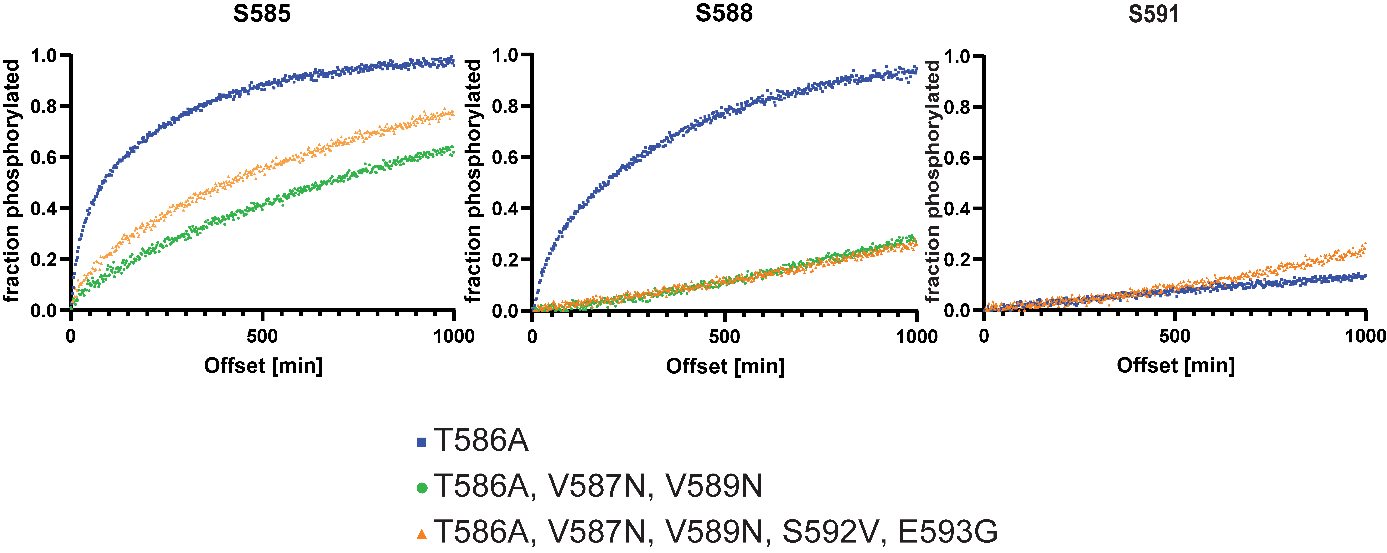


**Figure S10**. Comparison of the phosphorylation kinetics of S585, S588 and S591 in the wild type sequence (except for the T586A mutation) of the p63 PAD peptide (blue), the V587N, V589N mutant (green) and the V587N, V589N, S592V, E593G mutant (orange). The full length CK1δ kinase was used. Experiments with the T586A, V587N, V589N, S592V, E593G mutant were measured in triplicate, experiments with the T586A, V587N, V589N mutant and the wild type peptide (except for the T586A mutation) were measured in duplicate. The data shown represent one replicate.


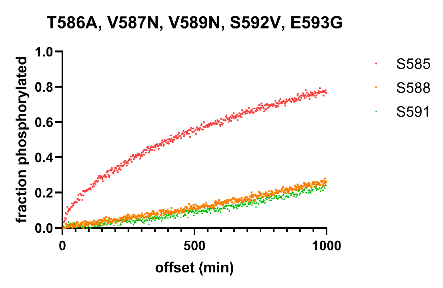

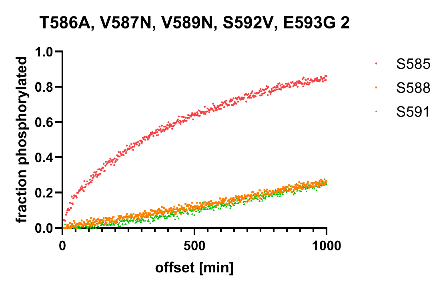

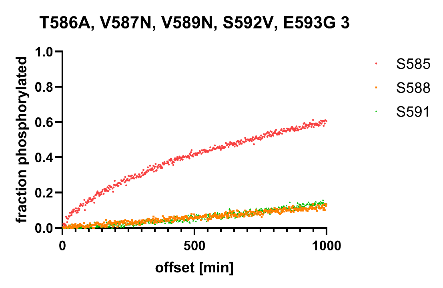


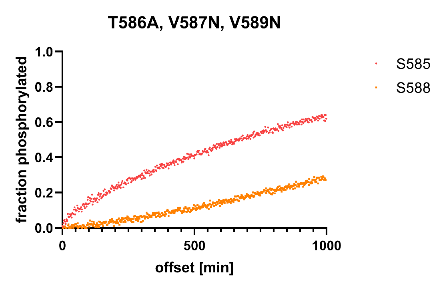

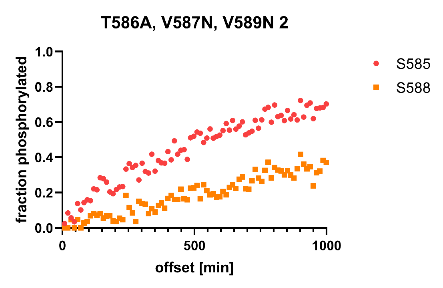


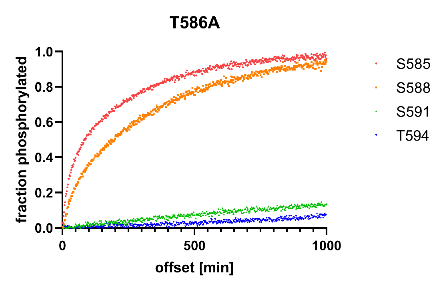

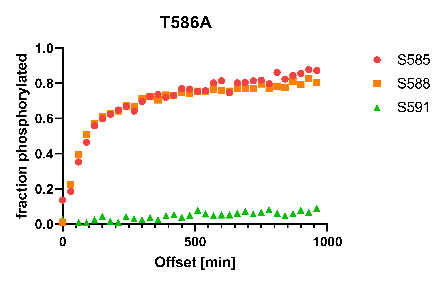


**Figure S11**. Replicates of the experiments shown in figure S2. Kinetics of the T586A, V587N, V589N, E593G were measured in triplicate. The kinetics of the T586, V587N, V589N and the wild type (except for the T586A mutation) peptides were measured in duplicate.


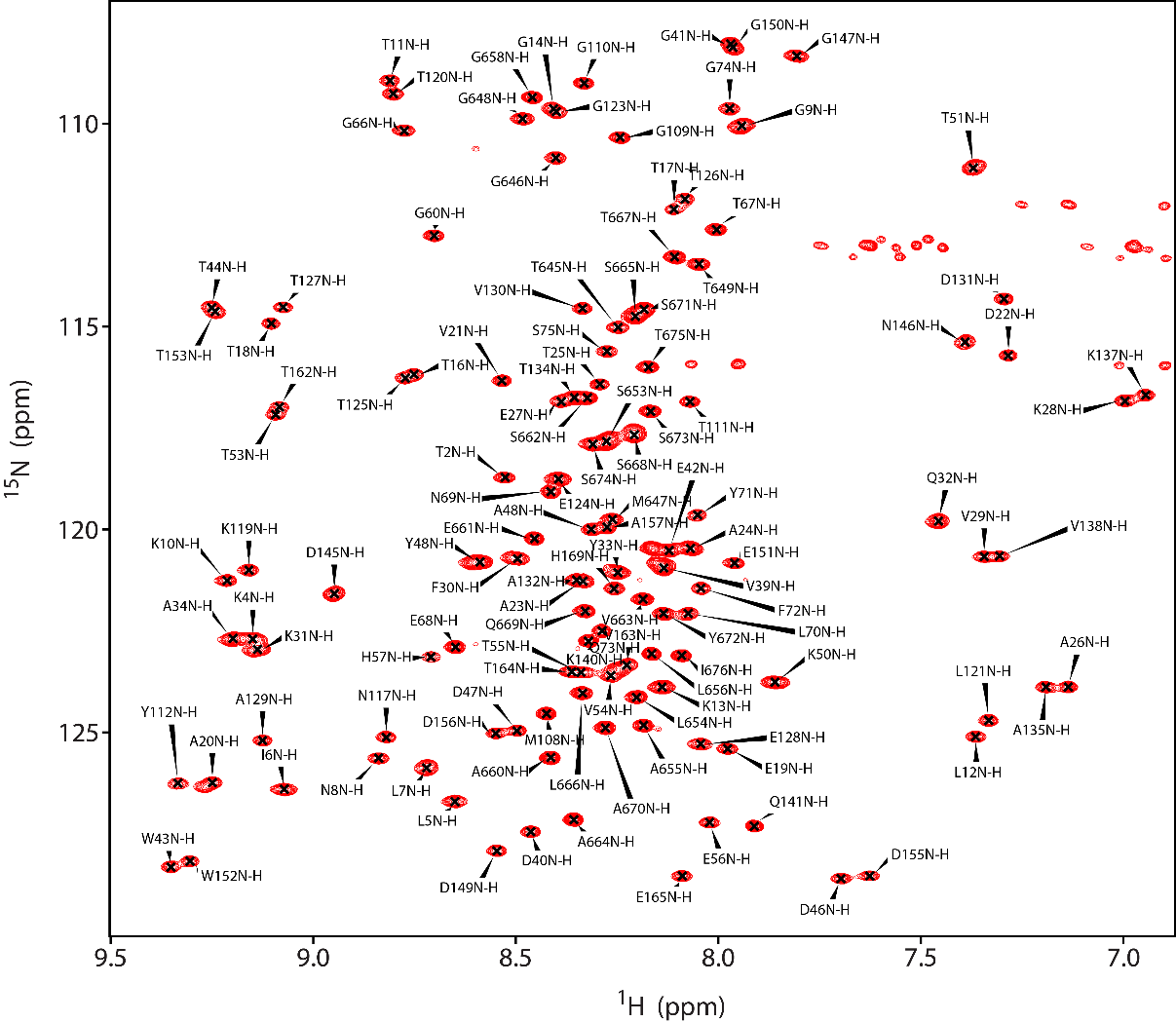
**a**

**
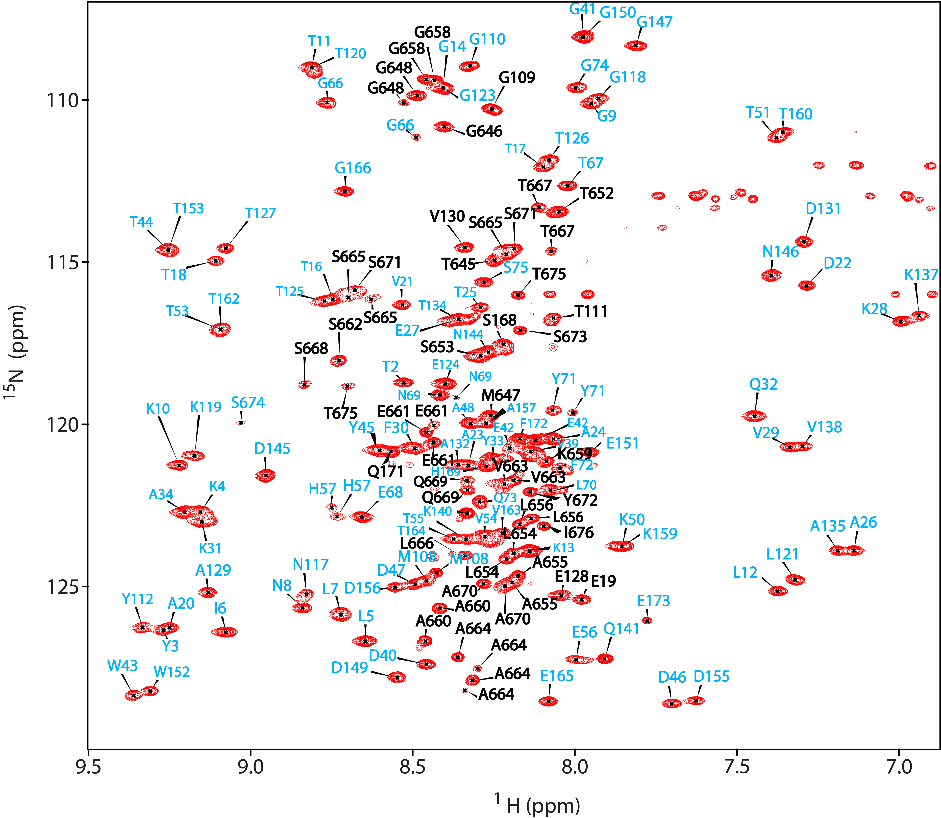
**

**b**

**Figure S12** (**a**) Assigned spectrum of the GB1-PER2-GB1 protein in the non-phosphorylated form and (**b**) phosphorylated with a 1:500 Ck1δ:substrate ratio over 24hrs. The resonance assignments for GB1 are indicated in blue, the assignments for the PER2 peptide in black.


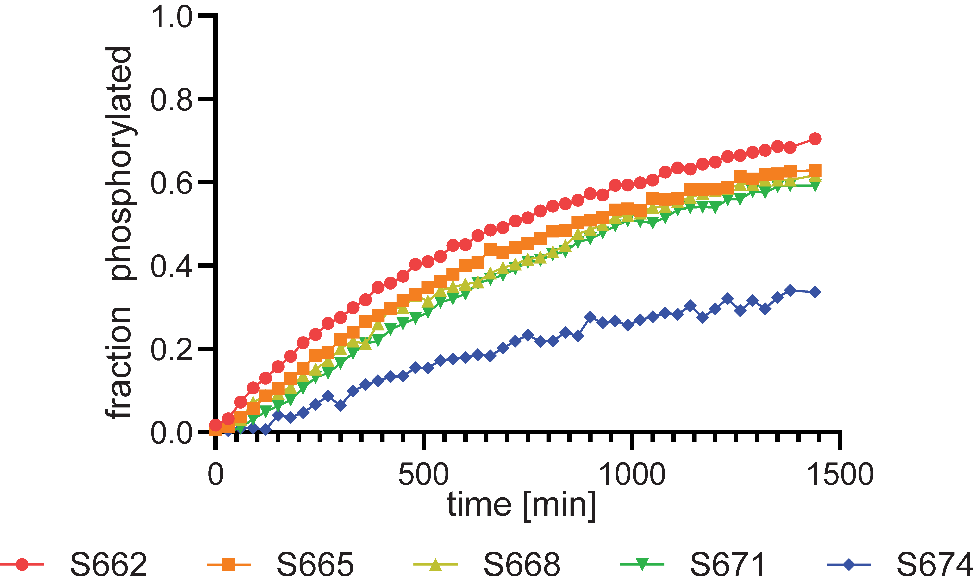


**Figure S13**. The phosphorylation kinetics of the human PER2 peptide. A C670A mutant was used to avoid problems with oxidation. Same experiment as reported in Fig.5a but with a 250:1 substrate:CK1δ ratio.


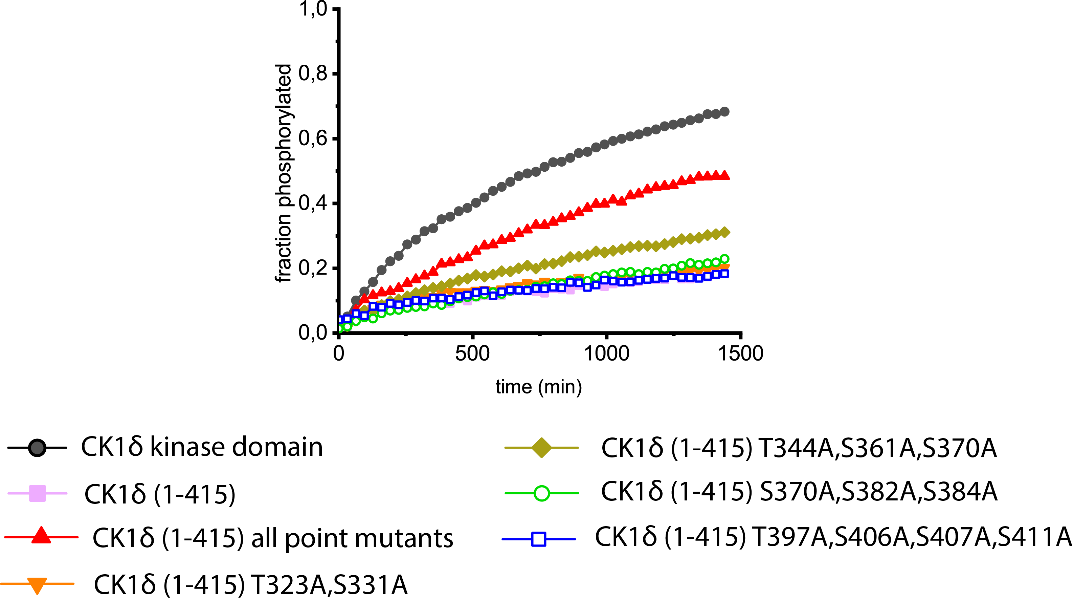


**Figure S14**. Replicate of the data shown in Fig 5b.


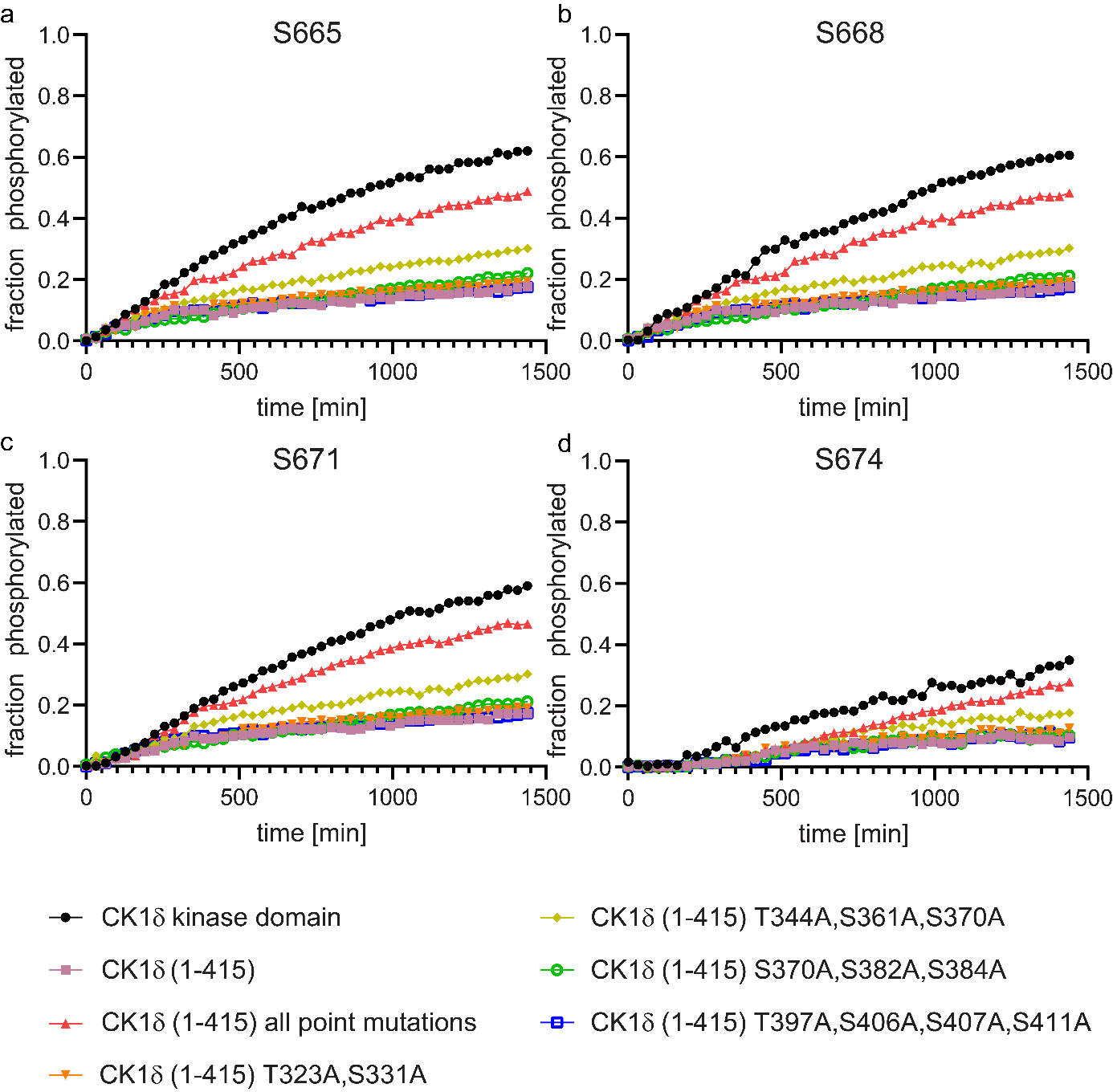


**Figure S15**. Comparison of the phosphorylation kinetics of (**a**) S665, (**b**) S668, (**c**) S671 and (**d**) S674 with the isolated CK1δ kinase domain, full length kinase and the indicated mutants. Experiments were measured in duplicate, the data shown represent one replicate.


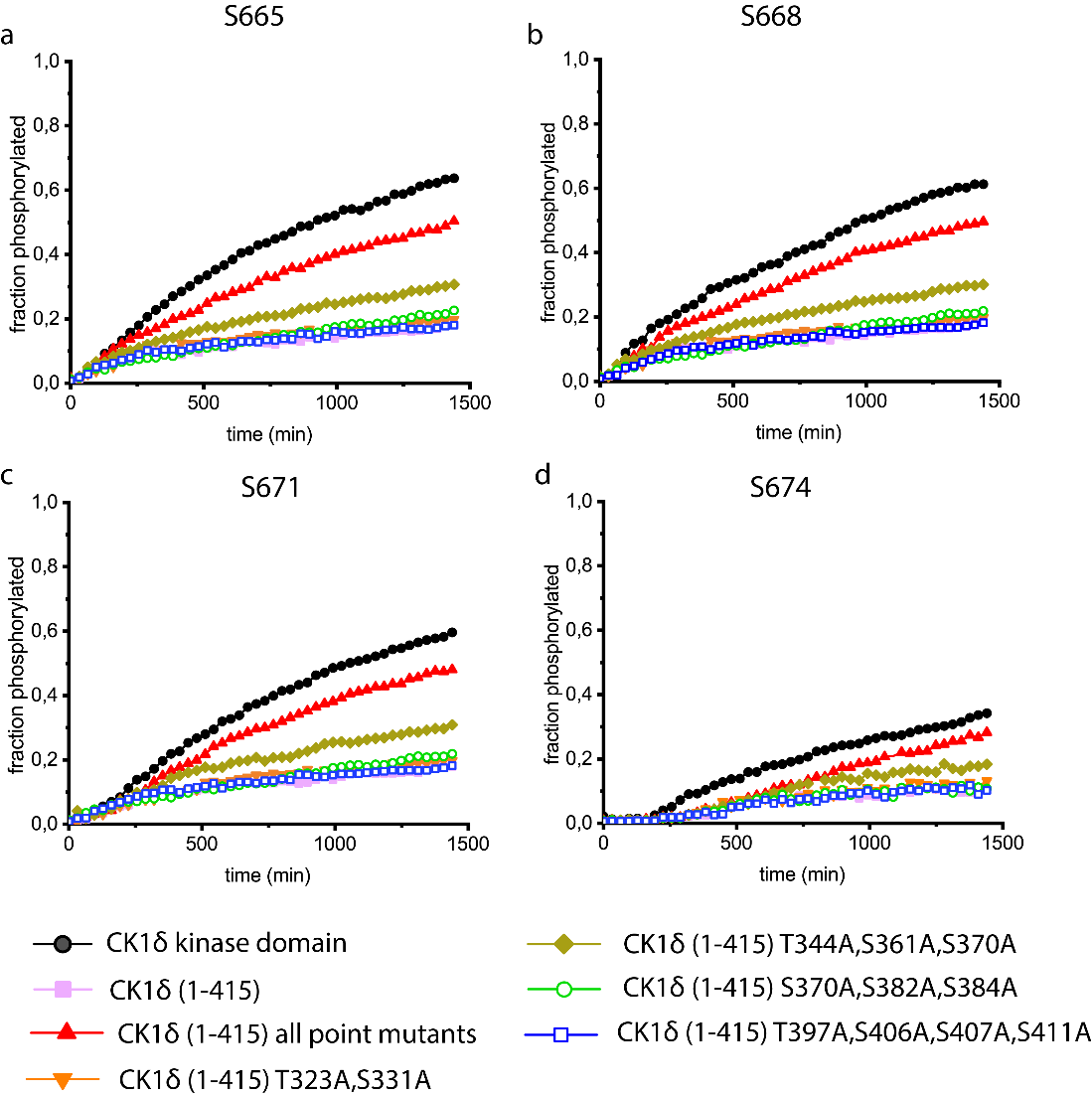


**Figure S16**. Replicate of the data shown in Figure S15.
